# Biliverdin reductase B as a new target in breast cancer

**DOI:** 10.1186/s13058-025-02147-x

**Published:** 2025-10-16

**Authors:** Natalia Marchenko, Natasha M. Nesbitt, Evguenia Alexandrova, Julie A. Reisz, Angelo D’Alessandro, Joonhyuk Suh, Stan Uryasev, Lisa Pennacchia, Wadie F. Bahou

**Affiliations:** 1 Department of Pathology, Stony Brook University, Stony Brook, NY 11794 USA; 2 Blood Cell Technologies, JLABS@NYC, 101 6th Avenue, New York, NY 10013 USA; 3 Department of Biochemistry and Molecular Genetics, University of Colorado, Aurora, CO 80045 USA; 4 Department of Applied Mathematics and Statistics, Stony Brook University, Stony Brook, 11794 USA; 5 Department of Medicine, Stony Brook University, Stony Brook, NY 11794 USA

**Keywords:** Redox homeostasis, Heme metabolism, HER2 positive breast cancer, Transferrin receptor

## Abstract

**Background:**

Enhanced metabolic and mitochondrial activity inherent in actively proliferating cancer cells is associated with intracellular redox imbalance that impacts cellular viability. To restore redox homeostasis cancer cells evolve to activate redox protective mechanisms. This differential activation of redox defense pathways compared to normal cells provides a therapeutic window for novel targeted therapies in cancer. Although heme metabolism emerges as a crucial regulator of redox homeostasis and iron metabolism in cancer cells with frequent alteration in breast cancer, it remains largely unexplored, and no targeted translational approaches have been developed. Heme-regulated redox homeostasis is coordinately maintained through biosynthetic and degradation pathways. As a byproduct of TCA cycle, cytotoxic heme is initially derivatized by heme oxygenases and progressively metabolized to the potent antioxidant bilirubin by two non-redundant biliverdin reductases, BLVRA and BLVRB. BLVRB overexpression has been observed in breast cancers, although its function in breast cancer pathogenesis remains unknown.

**Methods:**

CRISPR/Cas9 deletion of BLVRB in multiple breast cancer cell lines demonstrated its profound effect on intracellular redox state and cell proliferation in vitro and in xenograft models. Integrated proteomic, metabolomic, and lipidomic studies identified and validated BLVRB–mediated adaptive metabolic responses required for breast cancer cell cytoprotection.

**Results:**

We have established BLVRB as a requisite component of the pro-survival redox defense mechanism in breast cancer cells. Targeted deletion of BLVRB induces reductive stress, leading to alterations in endoplasmic reticulum proteostasis and lipid composition. These defects impact plasma membrane functionality and endosomal recycling of multiple oncogenic receptors, such as HER2 and transferrin receptors.

**Conclusions:**

These data collectively identify BLVRB as a novel metabolic target in breast cancer, distinct from other redox-regulating pathways. This study, along with our recent progress in developing novel specific BLVRB inhibitors, offers a unique translational opportunity for targeted therapies in personalized breast cancer medicine.

**Supplementary Information:**

The online version contains supplementary material available at 10.1186/s13058-025-02147-x.

## Background

Heme (Fe^[2+]^-protoporphyrin IX) plays an essential role in cellular redox homeostasis by functioning as a prosthetic group for phylogenetically distinct hemoproteins involved in diverse biological processes such as gas transport/exchange, catalysis, and the electron transport chain (ETC). An intact ETC comprised of hemoproteins is a requisite component of functional mitochondrial bioenergetics in malignant cells, and alterations in heme metabolism are frequently observed in cancer [1]. Metabolically active, proliferating cancer cells exhibit exaggerated dependence on energy production, display increased activity of heme-containing proteins and heme exporters [2], and develop redox-regulated adaptive mechanisms and dysregulated pathways that may lead to resistance to biologic- and chemotherapeutic-targeted therapies [3, 4]. Free cellular heme is highly reactive and regulates redox reactions due to its iron (Fe^2+/3+^) redox state, thereby generating reactive oxygen species (ROS)-associated cytoprotection loss and exaggerated cellular stress responses. Although previously published reports support the feasibility of targeting heme metabolism for cancer treatment [2, 5–7], the complex and context-dependent roles of heme metabolism in cancer have yielded limited therapeutic applicability.

**Fig. 1:**
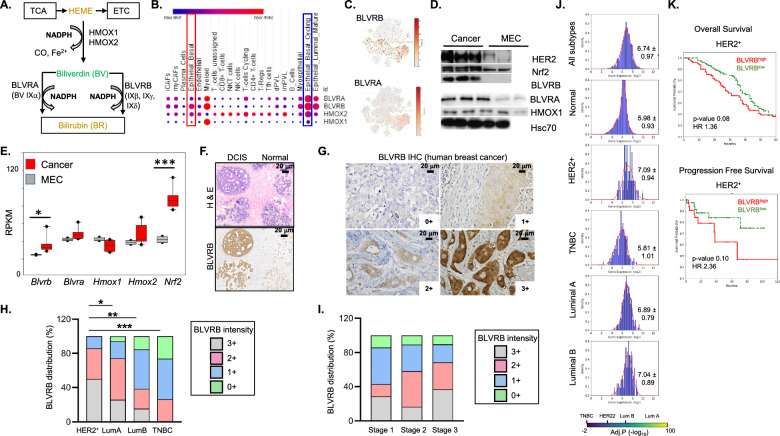
BLVRB is developmentally expressed in breast cancer and correlates with tumor progression and prognosis. **A** Schema shows the heme cofactor (Fe^[2+]^-protoporphyrin IX) as a critical component of hemoproteins (including the ETC, electron transport chain) that is generated as a TCA cycle byproduct from glucose and glutamine one-carbon pools (synthetic pathway), with sequential derivatization (degradation pathway) to the potent BR antioxidant by heme oxygenases (HMOX1, HMOX2) and two non-overlapping biliverdin reductases (BLVRA and BLVRB) with distinct substrate specificities and NAD(P)H as cofactor. BLVRA specifically utilizes BV IXα as substrate, while BLVRB uses non-IXα BVs (IXγ, IXδ, IXβ). **B**, **C** Single-cell RNAseq data extracted from human breast cancer data sets [20] delineate relative expression patterns of heme degradation pathway genes (*HMOX1*, *HMOX2*, *BLVRA*, *BLVRB*) by cell cluster; note the enrichment of *BLVRB* (and *BLVRA*) in cycling epithelial basal (blue rectangles) and epithelial basal (red rectangle), with generally enhanced *BLVRB* expression across all cell clusters (*Panel C* t-SNE plots; see Supplementary Fig. 1 for cluster definitions); scale bar is shown. **D**, **E** Murine epithelial cells (MEC) or mammary tumor cell lines were derived from MMTV/*ErbB2* mice [80], followed by immunoblots (*Panel D*), or RNA quantification (*Panel E*); data from *Panel E* are the RPKM (reads per kilobase per million) mean ± SEM for MEC (N = 2) or mammary tumor (N = 6) isogenic lines; *P*-values *p < 0.05, ***p < 0.001 by unpaired t-test. **F** Immunohistochemical (IHC) staining shows a progressive increase in BLVRB intensity during the transition from the normal mammary ducts (Normal) to DCIS within individual human breast cancer specimen (*lower panel*); parallel H & E staining is shown (*upper panel*); **G–I** BLVRB IHC staining in invasive human breast cancer is heterogeneous and displays nuclear, cytoplasmic, and membrane localization across specimens; staining intensity quantification in *Panel G* was applied to determine the distribution of BLVRB staining by subtype (HER2^+^ [N = 13], Luminal A [N = 47], Luminal B [n = 14], Triple Negative [TNBC, N = 22, *Panel H*], or by stage [Stage 1 [N = 9], Stage 2 [N = 68], Stage 3 [N = 22, *Panel I*]. *P*-values (*Panel H*) were calculated by unpaired t-test *p < 0.05, **p < 0.01, ***p < 0.001. In *Panel I*, BLVRB staining intensity correlates with advanced tumor stages as determined using the non-parametric Kendall’s τ-b coefficient (R = 0.22, *p*value < 0.006). **J** *BLVRB* RNA expression was extracted from human breast cancer TCGA (N = 1,082), or adult benign mammary samples (GTEx, N = 7,582) data sets, followed by data normalization for determination of relative abundance by breast cancer subtype. Histograms show *BLVRB* abundance (Log_2_) distributions, along with mean ± SEM; note the highest *BLVRB* expression in HER2^+^ (mean 7.04 ± 0.94), and lowest expression in normal (mean 5.98 ± 0.93) and TNBC (5.81 ± 1.01)l; the scale bar shows adjusted *p*-values (-log_10_) by cancer subtype (relative to normal mammary), calculated by nonparametric Mann–Whitney U test. **K** Kaplan–Meier curves using aggregated clinical data from TCGA and METABRIC datasets (N = 3,062) demonstrate trend of worse overall survival (HR 1.36, *p*-value 0.08) and progression-free survival (HR 2.36, *p*-value 0.1) in HER2^+^ breast cancer subtype in *BLVRB*^*high*^ cohorts

The balance of heme-regulated redox homeostasis is coordinated by heme synthetic and degradation pathways that represent functionally coupled bioenergetics systems that provide energy and cancer cell protection from metabolic stress [8]. Heme biosynthesis is a cataplerotic reaction that utilizes TCA (tricarboxylic acid)-derived carbon in a pathway linking TCA substrates (glucose and glutamine) to the heme degradation pathway [heme → biliverdin (BV) → bilirubin (BR), Fig. 1A]. The first step of heme degradation is catalyzed by heme oxygenases (HMOX1, HMOX2), resulting in the release of iron, carbon monoxide, and BV [1], followed by BV to BR conversion that occurs by two non-redundant biliverdin reductases (BLVRA [biliverdin IXα reductase) and BLVRB (biliverdin IXβ reductase]) that display non-overlapping redox substrates and limited structural homology (reviewed in [9]). BLVRB and BLVRA function in NADP(H)-dependent catabolic processes coupled to cellular antioxidant functions [8, 10]. BLVRA retains specificity for the predominant BV Ixα in adults [11], while BLVRB is promiscuous, catalyzing the reduction of non-IXα BVs (IXβ, IXγ, IXδ) [11–14], flavins [15], pyrroloquinoline quinones [16], and ferric ion [17]. BR is a lipophilic tetrapyrrole retaining potent antioxidant capacity that protects cells from 10,000-fold excess of H_2_O_2_ [9, 10], proposed as a redox-regulated cytoprotective mechanism beyond the glutathione (GSSG/GSH) couple [10]. Previous work in pluripotent stem cells (iPSCs) identified BLVRB in a bioenergetically-coupled pathway that maintains redox homeostasis and cytoprotection, with associated defects in glutamine TCA entry and ROS accumulation [18].

In this manuscript, we provide the first evidence for BLVRB as a critical, non-redundant regulator of redox homeostasis and survival in breast cancer cells. Heme oxygenases [2, 5–7] and BLVRA [10, 19–23] have been previously characterized in cancer progression and/or as potential cancer therapeutic targets, although limited evidence exists for BLVRB as a validated cellular cancer target independent of the HMOX/BLVRA axis [24]. While BLVRB overexpression has been observed in various malignancies [24–30], including breast cancers [31–34], its function in cancer pathogenesis has yet to be established. We now demonstrate that BLVRB functions as a spatially restricted antioxidant essential for redox homeostasis and cytoprotection of BLVRB-expressing cancer cells. Metabolic perturbations in genetically targeted BLVRB-deficient cells converge with the unfolded protein response (UPR), resulting in exaggerated endoplasmic reticulum (ER) stress. This stress leads to altered phospholipid metabolism, impairing membrane function and the trafficking of oncogenic receptors like HER2 and the transferrin receptor (TfR). These findings identify BLVRB as a unique cellular target in breast cancer, with putatively expanded applicability in cancer subtypes displaying BLVRB overexpression.

## Methods

### Materials and cell lines

Human breast cancer cell lines ZR-75–30, BT474, SKBR3, MDA231, MCF7, T47D and MCF10A were purchased from American Type Culture Collection (ATCC, Manassas, VA). SKBR3/BLVRB^+/+^ (*BLVRB*^+*/*+^) and SKBR3/BLVRB^−/−^ (*BLVRB*^*−/−*^) cells were cultured in McCoy’s 5A medium, while T47D, BT474, MCF7, and MDA-MB-231 were cultured in RPMI medium, all supplemented with 10% FBS, 100 U/mL penicillin G/Streptomycin and 1 µg/mL amphotericin B. Murine mammary cancer cell lines and mammary epithelial cells were generated from MMTV/*ErbB2* mice as previously described [35].

NADPH (nicotinamide adenine dinucleotide phosphate), DCPIP (2,6-dichlorophenolindophenol), and biliverdin IXα were purchased from Sigma, while biliverdin IXβ isomer was generated and purified from recombinant *Ps. aeruginosa* heme oxygenase as previously described [36].

### Generation of BLVRB loss-of-function and gain-of-function in vitro models

CRISPR/Cas9 was used to delete *BLVRB* from SKBR3 and T47D cells by transfecting cells with *BLVRB* double nickase plasmid (Santa Cruz Biotech cat# sc-405988), using TransIT-X2® Transfection Reagent (Mirusbio), followed by puromycin selection (6 g/ml) (Fisher) starting 48 h post-transfection. Selected cells were maintained in media supplemented with puromycin, and independent colonies were screened for BLVRB deletion by immunoblot analysis. For potential off-target effects associated with plasmid transfection, a non-targeting control plasmid (Santa Cruz Biotechnology, cat# sc-108060) was utilized as a negative control. Cellular proliferation assays were completed using trypan blue exclusion and automated cell counting (Invitrogen Countess 3).

Lentiviruses (Lv) expressing wild-type BLVRB (WT_BLVRB), the catalytically inactive BLVRB^S111L^ mutant (Mut_BLVRB), or an empty virus control (EV_GFP) were generated as previously described [37], and express green fluorescent protein (GFP) as bicistronic constructs driven by the cytomegalovirus (CMV) promoter and a puromycin-resistance cassette. BLVRB CRISPR/Cas9 derivatives of T47D (T47D/*BLVRB*^*−/−*^) were transduced with Lv/EV_GFP, Lv/WT_BLVRB, or Lv/Mut_BLVRB using multiplicity of infection (MOI) of 5, and at 72 h post-infection, cells were FACS-sorted based on GFP-positivity; GFP^+^ cells were > 95% across the genotypes. GFP^+^ cells were seeded in 12-well plates at a density of 5 × 10^3^ cells per well in triplicate for proliferation analysis at 48 h. Non-transduced (T47D/*BLVRB*^+*/*+^) cells and their corresponding BLVRB CRISPR/Cas9 derivatives (T47D/*BLVRB*^*−/−*^) were seeded in parallel as controls.

### Biochemical assays

Recombinant BLVRB used for enzymatic studies was expressed and purified as a glutathione *S*-transferase (GST) fusion protein in BL21(DE3) cells as previously described [36–38], and was > 95% pure (after carrier cleavage) as established by SDS-PAGE and densitometry. Cellular and enzymatic reduction assays were measured spectrophotometrically using a Cary 60 UV/Vis spectrophotometer. DCPIP reduction (DR) proceeded in a reaction containing 100 mM HEPES, pH 7.0, 100 μM NADPH, and 50 µM DCPIP at 25 °C. Reactions were initiated by the addition of RIPA-solubilized cellular lysates (50 µg), or pure BLVRB, and reductase activity was measured spectrophotometrically by following the decrease in absorbance at 600 nm corresponding to the reduction of DCPIP (ε = 20.7 mM^−1^ cm^−1^) [39]. Enzymatic conversion of BV IXβ to BR IXβ was assayed using 25 µM BV (determined using ε_650nm_ 15.5 mM^−1^ cm^−1^), 100 µM NADPH in 100 mM Tris–HCl pH 8.7, 1 mM EDTA at 25 °C, initiated by the addition of RIPA-solubilized lysates, and the reaction rate was determined by measuring the increase in absorbance at 450 nm corresponding to the production of BR using an extinction coefficient of 20.5 mM^−1^ cm^−1^. BLVRA activity in solubilized lysates was measured in a reaction mixture containing 50 mM Tris–HCl, pH 8.7, 100 μM NADPH, and 25 μM biliverdin IXα at 25 °C, and enzymatic activity was monitored following the conversion of biliverdin (ε_650nm_ = 14.3 mM^−1^ cm^−1^) to bilirubin (ε_450nm_ = 53 mM^−1^ cm^−1^) [11].

### Immunodetection analysis

Cells were lysed in 1X RIPA buffer (Rockland Immunochemical; Pottstown, PA) containing protease inhibitor cocktail (Millipore Sigma cat#P8340) and phosphatase inhibitor cocktail (Millipore Sigma cat#524,625). Cellular debris was removed by centrifugation at 10,000 × g for 15 min at 4 °C, and protein concentrations were determined by bicinchoninic acid (BCA) assay with bovine serum albumin (BSA) as standard [40]. Protein lysates were size-fractionated by SDS-PAGE [37], transferred to nitrocellulose membranes, and immunodetection was completed using sheep anti-BLVRB antibody (1:1000; R&D Systems; Minneapolis, MN cat#AF6568) followed by donkey anti-sheep antibody (1:2000; Jackson ImmunoResearch; West Grove, PA; cat#713–035-147); mouse anti-BLVRA (1:500; Santa Cruz; Dallas, TX cat#sc-393385) followed by goat anti-mouse antibody (1:2000; Jackson ImmunoResearch; West Grove, PA cat#115–585-003); goat anti-Transferrin Receptor (CD71; R & D Systems, Minneapolis, MN cat#AF2474) followed by mouse anti-goat antibody (1:2000; Jackson ImmunoResearch; West Grove, PA cat#205–032-176); mouse anti-HNE antibody (1:1,000; Thermo Fisher Scientific, cat#MA5-27,570) followed by sheep anti-mouse antibody (1:10,000 dilution), mouse anti-heme oxygenase I (1:1000; Novus Biologicals; Centennial, CO cat#NBP1-97,507) followed by sheep anti-mouse antibody (1:10,000); mouse anti-heme oxygenase 2 (1:100; Santa Cruz; Dallas, TX cat#sc-17786) followed by sheep anti-mouse antibody (1:10,000) mouse anti-actin antibody (1:1,000; Thermo Fisher Scientific; Waltham, MA cat#MA1-744) followed by sheep anti-mouse antibody (1:10,000; GE Healthcare; Chicago, IL cat#NA9310) Hsc70 (#sc-71270) Hsp90 (#sc-69703) (all from Santa Cruz Biotechnology); NQO1 (#62,262), PERK, BIP, CHOP (ER Stress Antibody Sampler Kit #9956), PARP (#9542), HER2, pHER2(Y1221/Y1222) (HER/ErbB Family Antibody Sampler Kit #8339), Pan-R-Tyr (Phospho-Tyrosine (P-Tyr-1000) MultiMab® Rabbit mAb mix #8954), EE1A, caleolin, clathrin, RAB5A, RAB7 (Endosomal Marker Antibody Sampler Kit #12,666) all from Cell Signaling. Immunodetection was completed by enhanced chemiluminescence using Luminata™ Forte Western HRP substrate (Millipore Sigma; Burlington, MA) and visualized using a C-DiGit® blot scanner (LI-COR; Lincoln, NE). All immunoblots were repeated at least two times.

Cellular quantification of BLVRB was completed by ELISA using lysates (diluted 1:5 in diluent), and 50 µL aliquots were assayed in duplicate using a sandwich ELISA kit (Abcam), quantified to a standard control curve displaying an R^2^ = 0.999. The lipid peroxidation adduct malondialdehyde (MDA) was quantified by ELISA (Abcam), using solubilized cellular lysates (200 µL) prepared by homogenization at 4 °C in MDA lysis buffer. After centrifugation, samples were incubated for 60 min at 95 °C with thiobarbituric acid (TBA, 0.02% *w*/*v*), and the appearance and quantification of the MDA-TBA adduct was determined by absorption at 532 nm, quantified to a standard curve run in parallel. Intracellular ROS was quantified by ROS-GLO cell-based assays (Promega); cells (1 X 10^4^) were propagated in 96-well plates until > 90% confluent, and sequentially incubated with hydrogen peroxide substrate solution (6 h at 37 °C to generate the luciferin precursor), followed by recombinant luciferase detection reagent (20 min at 25 °C) prior to luminescence quantification using a Spectramax M5 microplate reader (Molecular Devices).

### Flow cytometry

Cultured cells were harvested by incubation with EDTA, pelleted (1500 r.p.m. for 10 min), and resuspended in PBS for immunophenotyping, or fixed and permeabilized (70% ethanol/0.25% triton X) for cell cycle analysis using [20 μg/ml propidium iodide and 10 μg/ml RNase A in PBS] at 4 °C for 30 min in the dark, prior to cell cycle data acquisition. Cell-surface immunophenotyping was completed as previously described using fixed (4% paraformaldehyde for 15 min at 4° C) and non-permeabilized cells [41], incubated at 4° C for 15 min in the dark with FITC-conjugated anti-CD71 (transferrin receptor, 0.65 µg/mL) or PE (phycoerythrin)-conjugated anti-HER2 (0.65 µg/mL) MAbs. Intracellular ROS accumulation was quantified using the cell-permeant fluorogenic probe CellROX Red [500 nM, 30 min at 37 °C] (Life Technologies; Carlsbad, CA). Flow cytometric quantification was completed by data acquisition of 10,000 events using logarithmic gain settings for light scatter and fluorescence detection, applying isotype-matched IgG controls for gate delineation (CytoFLEX; Beckman Coulter; Brea, CA); data were analyzed using Kaluza Flow Cytometry Analysis Software (Beckman Coulter; Brea, CA).

### Q-PCR

Cellular RNA was isolated using Trizol, followed by quantification and characterization using a 2100 Bioanalyzer (Agilent Technologies). Transcript abundance was determined using fluorescence-based real-time Q-PCR (polymerase chain reaction) technology (Opticon 2 System, Bio-Rad, Hercules, CA) as previously described [41–44], determined from triplicate assays performed in parallel (2 ng input RNA), normalized to β-*actin* (oligonucleotide primers are provided in Supplementary Table 2).

### Immunofluorescence

Cells were grown on chamber slides until ~ 70% confluent, media was aspirated and cells were washed X 3 in PBS; following methanol fixation (-20 °C for 10 min), cells were permeabilized with 0.2% Tween 20 in PBS for 10 min, and incubated with blocking buffer [10% normal horse serum (NHS) and 0.1% Tween 20 in PBS] for 1 h at 37 °C. Cells were then stained with rabbit anti-BLVRB (1:1000, Sigma), mouse anti-HER2 (1:1000; #191,924, Novus Biologicals), and mouse anti-HMOX2 (1:200; Santa Cruz Biotechnology) for 2 h at 37 °C, and then washed X3 with PBS. Alexa fluor-labeled goat anti-rabbit or goat anti-mouse secondary antibodies (Molecular Probes) were then added at 1:500 dilution for 30 min at 37 °C. The cells were then washed, counterstained with Hoechst (2 μg/mL), mounted with Prolong gold (Molecular Probes), and mounted onto coverslips before image capture using a Zeiss 910 confocal microscope. Acquisition of two-color images was performed in sequential scanning mode to minimize spectral bleed-through artifacts.

### Immunohistochemistry (IHC)

Human breast cancer tissue microarrays (Tissue array #BR2082c) were deparaffinized in xylene, sequentially rehydrated using graded alcohol dilutions, and antigen retrieval was completed using citrate buffer at 120 °C for 10 min. Endogenous peroxidase activity was quenched with hydrogen peroxide, followed by overnight incubation with primary rabbit BLVRB antibody (Sigma #HPA041937, 1:1000) at 4 °C, and immunodetection using the biotinylated horse secondary antibody (Vector Laboratories), visualized using 3,3′-Diaminobenzidine (DAB) chromogen (EnVision 2-component system, Agilent, Santa Clara, CA); negative controls included the isotype-matched immunoglobulin. Hematoxylin was used for counterstaining, and individual samples were scored [no staining (0), weak staining (1 +), moderate staining (2 +), strong staining (3 +)] by a surgical pathologist with no knowledge of sample identity. Oil Red O staining was performed using Oil Red O stain kit (#NC9870866 StatLab) according to the manufacturer’s protocol.

### Proteomic analyses and pathway reconstruction

Cells (5 × 10^6^/condition/genotype) were lysed (5% SDS, 50 mM TEAB (triethylammonium bicarbonate), pH 8.5) and reduced (10 mM DTT at 55 °C for 30 min), followed by alkylation in 25 mM iodoacetamide, prior to loading onto an S-Trap mini cartridge (Protifi). Samples were then digested with trypsin (20 µg) in 50 mM TEAB in a humified incubator overnight at 37 oC, sequentially eluted (50 mM TEAB, 0.2% formic acid, and 50% acetonitrile), and lyophilized, before resuspension (200 µL 0.1% trifluoracetic acid, TFA), and desalting on an HLB reverse phase cartridges (Waters). Samples (in duplicate) were eluted (20% and 50% acetonitrile), and parent peptide mass, collision-induced fragment (CID) mass, and peptide abundance values were obtained by liquid chromatography-electrospray ionization tandem mass spectrometry (LC–MS/MS) using an orbital trap instrument (Thermo Q-Exactive HF). Data files were acquired using Xcalibur software, and peptide alignments and quantitation were performed using Proteome Discoverer (*version* 3.1, Thermo Scientific). Protein false discovery rates (FDR) were binned at 0.01 and 0.05 FDR, while the peptide-spectrum mass (PSM) was set to 0.01; mass resolution search cutoffs were 10 ppm and 0.05 Daltons (respectively), and two missed tryptic cleavages were allowed for peptide identification [putative modifications included static cysteine derivitizations, variable deamidation (NQ), water loss (ST), oxidation (M), and phosphorylation (STY)]. Log_2_ abundance data were normalized using TMM (Trimmed mean of M-values), and data processing was completed using Bioconductor R statistical package (*v 3.7)* [45], incorporating a stringent filtering step requiring expression across all samples for integrated comparisons [46]. Differential expression (DE) analyses were performed by fitting a generalized linear model (glm) and conducting likelihood ratio tests [41, 44], using normalized (log_2_-transformed) data and clustering (on Euclidean space) for cross-group comparisons. Hierarchical clustering was performed using Euclidean distance between samples, and applying dissimilarities by Ward’s method. For all analyses, statistical significance (*p* < 0.05) was adjusted for false discovery using Benjamini–Hochberg methodologies [47]. The imputed abundance list ranked by significance [(log_2_ fold-change)*(-log_10_ adjusted *p*-value)] was used for pre-ranked GSEA against curated Hallmark gene (Molecular Signature Database (MSigDB, *ver* 7.0) [48]. Sources for construction of the interactive networks included well-curated database (Reactome, KEGG, Human PPI), which were trained and validated using a naïve Bayes classifier.

### Metabolomic analyses

Cells (2 × 10^6^/condition/genotype) were grown in DMEM (with or without 10% FBS) for 18 h, washed and detached, followed by cell lysis and harvesting (for targeted metabolites), or supernatant harvesting in 1:25 dilution of lysis solution (5:3:2 MeOH:Acetonitrile(ACN):H_2_O). Samples were vortexed for 30 min at 4 °C, precipitated by centrifugation (10 min at 18.000 × g, 4 °C), and supernatant metabolites were resolved over a Kinetex C18 column (2.1 × 150 mm, 1.7 μm, Phenomenex) using a Vanquish UHPLC system with injections of 15 µL volumes (supernatant extracts) or 10 µL volumes for cellular extracts. The UHPLC was coupled to a high-resolution Q Exactive mass spectrometer (Thermo Scientific). Samples were injected for positive and negative ion mode using a 5 min gradient at (450 µL/min from 5 to 95% of ACN/0.1% Formic Acid in Water/0.1% Formic Acid, positive mode), and (95% ACN/5% water/1 mM ammonium acetate in 5% ACN/95% water/1 mM ammonium acetate, negative mode). Raw files were converted to mzXML file format using Raw converter [49, 50] and technical replicates were used to control technical variability. Peak annotation and integration were performed using MAVEN integrated with KEGG database, and metabolic enrichment analyses were completed using MetaboAnalyst (*Version* 6.0) applying the hypergeometric test for pathway over-representation analysis [51]. False discovery was minimized using the adjusted Holm *p*-value (Holm-Bonferroni method).

### Lipidomic analyses

Lipidomic analyses were completed using extracts and supernatants (vide supra) obtained using a Vanquish UHPLC system coupled to a Q Exactive mass spectrometer (Thermo Fisher Scientific). The samples were randomized and resolved across a 2.1 × 30 mm, 1.7-µm Kinetex C18 column (Phenomenex) using a 5-min reversed-phase gradient as previously described [52]. Technical replicates were included to assess quality control. Lipid assignments and peak integration were performed using LipidSearch v 5.0 (Thermo Fisher Scientific). Statistical and pathway analyses, including linear discriminant analyses (LDA), principal component analysis, hierarchical clustering analysis, and heat map generation based on LDA results, were performed with MetaboAanalyst 6.0 analyses and BioPAN [53]. Violin plots were generated in RStudio 2023.06.2 Build 561.

### Xenograft studies

Female, 5-week-old athymic nude mice were procured from Jackson Laboratory (Stock 002019). Because breast cancer is rare in men, only female mice were used to address sex as a biological variable. All animals received care under IACUC and institutional guidelines (IACUC# 921610). On the day of implantation, cells were dissociated from flasks using 0.25% trypsin/1 mM EDTA, trypsin was neutralized with RPMI/10% FBS, and cells were collected and concentrated by centrifugation at 300 x g for 5 minutes. Media was aspirated and cells were resuspended in 50:50 Cultrex Basement Membrane Extract (BME):RPMI (no supplementation) at a concentration of 2 x 10^7^ cells/mL; viability using trypan blue exclusion was >97%. A fixed volume (100 µL, 2 X 10^6^ cells) was injected into the right hind flank of each animal. For hypothesis testing at a 0.05 (1-sided) significance level, the estimated power of detecting a difference between *BLVRB*^+*/*+^ and *BLVRB*^*-/-*^ mice with N=10/cohort is >95%. Mice were assigned to one of two implantation groups: *BLVRB*^+*/*+^ and *BLVRB*^*-/-*^, and after implantation, tumor volume was measured blindly twice weekly for 24 days (total of 7 measurements). Throughout the study, general animal well-being was monitored along with animal weights on the same schedule as those for tumor volume, with no evidence for weight loss, general discomfort, pain, or inactivity. Tumors were measured in two dimensions using calipers, and volume was calculated using the formula: tumor volume (V, mm^3^) = 0.5 X *w*^*2*^ x *l*, where *w* = width and *l* = length (in mm). Animals were euthanized when the tumor reached a volume of 3×10^3^ mm^3^, the maximal tumor size according to institutional guidelines (IACUC# 921610).

### Clinical bioinformatic studies

Databases used in this study include breast cancer cohorts from METABRIC (N = 1,980) [54], Cancer Genome Atlas TCGA (N = 1082) [55], or normal individual GTEx datasets (N = 7,582) [56]. RNAseq expression analyses (*BLVRB*, *BLVRA*, and *HMOX*) were from TCGA and GTEx, while Kaplan–Meier plots were generated from combined TCGA and METABRIC datasets (N = 3,062); all data were batch-normalized and log_2_-transformation before analyses. TCGA and METABRIC data sets were merged using normalized expression data across both cohorts, and cross-data set validation was confirmed by the comparative distribution of the normalized gene expression data by Kolmogorov–Smirnov test. Data were analyzed on breast cancer subtypes, including HER2^+^, Basal, Luminal A, and Luminal B. The Mann–Whitney U test was used to compare the medians of gene expression in different breast cancer subtypes to gene expression in normal tissues. For survival analyses, breast cancer patients were divided into two groups (*BLVRB*^*high*^, *BLVRB*^*l*^*°*^*w*^) based on median *BLVRB* expression levels. Overall survival between high and low-expression cohorts was then compared within each breast cancer subtype. Survival curves were generated using Kaplan–Meier plots, and hazard ratios (HRs) were calculated using Cox proportional hazard regressions (delimited by subtype). ROC (receiver operating characteristic) plots quantifying response to HER2-targeted or 5FU-based therapies delimited by *BLVRB*^*high*^, *BLVRB*^*lo*^^*w*^ expression were generated using a biomarker assessment platform [57].

### Statistical analysis

Statistical comparisons were completed using ANOVA or Student’s t-tests (or their non-parametric counterparts if the normality assumptions were not met) at the significance level of *p* < 0.05. Simple linear regression was performed in GraphPad Prism (*version* 9.3.1) for correlation analyses.

## Results

### *BLVRB* is expressed in epithelial cells involved in breast cancer pathogenesis

Extraction of heme degradation pathway (*HMOX1, HMOX2, BLVRB, BLVRA*) transcriptomic data from the NCI-60 cell line database confirmed enhanced *BLVRB* expression compared to *BLVRA* (or *HMOX1/HMOX2*) across the majority (53/60) of cancer cell lines of different origins. In the five breast cancer lines, the higher BLVRB expression was evident in Luminal A (MCF7, T47D) compared to triple-negative breast cancer (TNBC) cell lines (MDA-MB- 231, HS-578 T, BT549) (Supplementary Fig. 1A). Restricted expression patterns were evident using human single-cell transcriptomic data from breast cancer biopsies [58], where heme degradation pathway genes were selectively expressed in myeloid, cycling epithelial cancer cells, and mature luminal cell clusters (Fig. 1B). Exaggerated *BLVRB* > *BLVRA* levels were evident across the heme degradation clusters, and BLVRB expression was enriched in cycling (compared to non-cycling) basal epithelial cells, a population implicated in mammary tumorigenesis (Fig. 1B, C and Supplementary Fig. 1B–D). To better understand the putative oncogenic functions of heme degradation pathway components during tumor initiation, we extended these observations to isogenic cell lines derived from previously generated benign mammary epithelial cells (MECs) and malignant tissues from MMTV/*ErbB2* mice [35, 59, 60]. Elevated BLVRB protein (Fig. 1D) and mRNA levels (Fig. 1E) were evident in cancer cells compared to MECs upon malignant transformation. In contrast, there were no statistically significant differences in *HMOX1*, *HMOX2*, or *BLVRA* mRNA expression comparing MEC to cancer cells (Fig. 1D, E). *BLVRB* induction (3.2-fold, *p* < 0.05) was comparable to that of the major antioxidant transcription factor *NRF2* (Nuclear erythroid factor 2, 3.1-fold) and consistent with the dysregulation of redox homeostasis during malignant transformation. This distinct expression pattern of *BLVRB* (compared to other heme degradation genes) mirrors the pattern observed in cycling stem cells [37].

### BLVRB expression correlates with breast cancer progression and prognosis

Immunohistochemical staining (IHC) of stage-delimited human breast cancer demonstrated heterogeneous BLVRB staining across normal mammary glands, with progressive increase during the transition from benign mammary epithelial cells to ductal carcinoma in situ (DCIS) (Fig. 1F). BLVRB distributional staining in invasive breast cancer specimens was variable, and localized to the nucleus, cytoplasm, and membrane (Fig. 1G). Extension of these observations using a human breast cancer tissue microarray (TMA, N = 73 samples) demonstrated significant heterogeneity in the intensity of BLVRB staining across breast cancer subtypes and stages. Notably, HER2^+^ breast cancers exhibited the greatest BLVRB staining, while TNBC (triple-negative breast cancer) tissues demonstrated the lowest (Fig. 1H, I). Pairwise comparisons identified statistically significant differences in BLVRB expression between HER2^+^ and TNBC (*p* = 0.0003), HER2^+^ and Luminal A (*p* = 0.02), and HER2^+^ and Luminal B (*p* = 0.006) subtypes. Across the samples, a statistically significant linear correlation was observed between elevated BLVRB expression and advanced tumor stages (R = 0.22, *p*-value = 0.006), consistent with a role in breast cancer progression. Comparable patterns were evident using RNAseq data from The Cancer Genome Atlas (TCGA) breast cancer (N = 1,082) [55] and Genotype-Tissue Expression (GTEx) datasets (adult benign mammary tissue, N = 7,582) [56], where *BLVRB* expression progressively increased from normal mammary tissue (mean = 5.98 ± 0.93) to breast cancer (all subtypes, mean = 6.74 ± 0.97, Fig. 1J). *BLVRB* expression was greatest in HER2^+^ subtypes (mean 7.09 ± 0.94) and not elevated in TNBC (mean 5.81 ± 1.01), results that recapitulates expression patterns using IHC (Fig. 1H) and breast cancer cell line expression analyses (Supplementary Fig. 1A). *BLVRA* expression patterns mirrored those of *BLVRB* (*i.e.* HER2^+^ [mean 6.38 ± 0.66] > TNBC [mean 5.31 ± 0.77]), although greatest mean expression was identified in Luminal B [mean 6.81 ± 0.80] subtype (Supplementary Fig. 2A). While *HMOX1* levels in aggregated breast cancer subtypes (mean 4.88 ± 1.01) were slightly elevated compared to normal tissue (mean 4.01 ± 1.97), no substantial difference was observed between HER2^+^ (mean 5.04 ± 0.88) and TNBC (mean 4.87 ± 1.09) subtypes. This contrasts with the significantly higher expression of *BLVRB* and *BLVRA* found in the HER2^+^ subtype compared to TNBC. (Fig. 1J, Supplementary Fig. 2A, B).

Although not reaching conventional statistical significance (*p* < 0.05), survival analyses in the TCGA and METABRIC cohorts (N = 3,062) demonstrated a trend toward poorer prognosis associated with elevated *BLVRB* expression in HER2-positive breast cancer, reflected in reduced 5-year overall survival (Hazard Ratio [HR] = 1.36, *p* = 0.08) and progression-free survival (HR = 2.36, *p* = 0.10) (Fig. 1K and Supplementary Fig. 2E). Divergent patterns were evident in *BLVRA*^*high*^ cohorts, which showed no prognostic effects in HER2^+^ cohorts, and improved 5-year OS in basal (TNBC, HR 0.67, *p*-value 0.03) and Luminal A (HR 0.72, *p*-value 0.01) cohorts (Supplementary Fig. 2C). *HMOX1* expression showed no OS differences in any subtype (Supplementary Fig. 2D). These aggregated data suggested divergent clinical and phenotypic characteristics between *BLVRA-* and *BLVRB*-expressing breast cancer cohorts, and a potential oncogenic BLVRB function in mammary tumorigenesis, particularly in the context of HER2^+^ breast cancer.

### BLVRB drives breast cancer cell proliferation

**Fig. 2:**
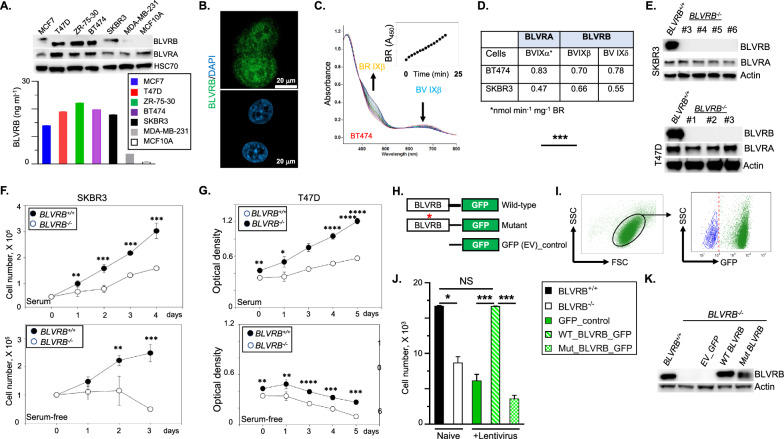
BLVRB promotes the proliferation of breast cancer cells. **A** Immunoblot (20 µg/lane) and quantitative ELISA show high BLVRB expression in SKBR3 (HER2 ^+^), BT474 and ZR-75–30 (Luminal B), and MCF7 and T47D (both Luminal A); low BLVRB expression in MDA-MB-231 (TNBC), and lowest/undetectable in non-tumorigenic MCF10A mammary epithelial cells. **B** Confocal microscopy of SKBR3 cells demonstrates diffuse cytoplasmic BLVRB expression, with a smaller component of cell membrane immunofluorescence; a size marker (20 µm) is shown. **C**, **D** BLVRB and BLVRA enzymatic activities (nmol min^−1^ mg^−1^) were determined using SKBR3 and BT474 lysates (50 µg) and 25 µM isomer-specific biliverdin Ixβ/IXδ (BLVRB) or biliverdin IXα (BLVRA); a representative UV/Vis scan using BT474 lysates demonstrates the time-dependent loss of BV IXβ with concomitant appearance of BR IXβ (*inset*). **E** CRISPR-mediated *BLVRB* depletion in SKBR3 (HER2^+^) and T47D (Luminal A) cells confirms BLVRB loss without affecting BLVRA levels; immunoblots were completed using 20 µg lysates/lane. **F**, **G** Cellular proliferation curves (5 X 10^4^/well) using SKBR3 (*Panel F* cell counts) or T47D (*Panel G,* MTT colorimetric determination (optical density (OD) absorbance at 550 nm) cells were completed under serum (*upper panel*) or serum-free conditions (*lower panel*), and presented as the mean ± SEM (N = 3—6 wells/genotype) from technical and biological replicates; *p*-values * < 0.05; ** < 0.01; *** < 0.001; **** < 0.00001 using unpaired t-test. **H**–**K** Schema (*Panel H*) shows GFP-expressing wild-type BLVRB (WT_BLVRB), the catalytically inactive BLVRB ^S111L^ mutant (Mut_BLVRB), or an empty virus control (EV_GFP) used to infect T47D/BLVRB^−/−^, followed by flow cytometric sorting (gates shown in *Panel I*) applied to study proliferation of 5 X 10^3^ cells/well (12 well plate, N = 3/genotype) at 48 h (*Panel J*); non-transduced T47D/*BLVRB*^+*/*+^ or T47D/*BLVRB*^*−/−*^ are shown as controls (*Panel J*); the corresponding immunoblots of genetically-complemented T47D/*BLVRB*^*−/−*^ cells were completed using 20 µg/lane (*Panel K*). Note the genetic complementation with WT_BLVRB, but neither mutant nor empty vector GFP controls. *p*-values in *Panel J* * < 0.05, *** < 0.001 were determined using unpaired t-test; NS – not significant

Immunoblot analysis and quantitative ELISA of human breast cancer cell lines revealed variation in BLVRB expression, with the highest expression in cell lines SKBR3 (HER2^+^), BT474 and ZR-75–30 (both Luminal B), and MCF7 and T47D (both Luminal A); BLVRB expression was minimal in MDA-MB-231 (TNBC), and lowest/undetectable in non-tumorigenic MCF10A mammary epithelial cells (Fig. 2A). BLVRA expression was detectable in all cells by immunoblot, but (unlike BLVRB) was also identified in non-tumorigenic MCF10A (Fig. 2A) and murine MECs (Fig. 1D). Confocal microscopy of BLVRB-expressing SKBR3 cells revealed a subcellular distribution of BLVRB in the cytoplasm and nucleus (Fig. 2B), consistent with the heterogeneous localization observed in human breast cancer tissue (Fig. 1G). Enzymatic activities in BT474 and SKBR3 cells established that BLVRB-specific activity was nearly identical between SKBR3 (0.66 nmol BR min^−1^ mg^−1^) and BT474 (0.70 nmol^−1^ BR min^−1^ mg^−1^) lysates, with slightly greater BLVRA specific activity in BT474 (0.83 nmol^−1^ BR min^−1^ mg^−1^) compared to SKBR3 (0.47 nmol^−1^ BR min^−1^ mg^−1^) lysates (Figs. 2C, D). Since BLVRA has ~ fourfold greater specific activity for its IXα substrate (~ 1300 nmol BR min^−1^ mg^−1^) than BLVRB for IXβ (~ 350 nmol BR min^−1^ mg^−1^) [36], these data imply exaggerated BLVRB reductase activity (compared to BLVRA) in both SKBR3 and BT474 cells.

For further characterization, we applied CRISPR-mediated *BLVRB* ablation in two distinct BLVRB-expressing cell lines, distinguished by HER2 expression: SKBR3 (HER2^+^) or T47D (HER2-negative, Luminal A) [61]. Independent clones from both cell lines confirmed loss of BLVRB with no reciprocal effects on BLVRA expression (Fig. 2E), and both *BLVRB*-ablated cell lines exhibited statistically significant decreased proliferation (Fig. 2F, G), which was accompanied by statistically-significant cell cycle effects, with SKBR3/*BLVRB*^*−/−*^ cells displaying a decrease in G1 (*p*-value < 0.01) and enhanced G2/M arrest (*p*-value 0.001) (Supplementary Fig. 3A). Nutrient deprivation is a common feature of the tumor microenvironment (arising from poor vascularization, high metabolic demand, and stromal cell nutrient competition). Under serum-free conditions designed to mimic the metabolic tumor microenvironment, proliferative loss in *BLVRB*-deficient cells was exacerbated in both cell line (Fig. 2F, G). Gene-delimited specificity of the targeting construct was confirmed using BLVRB genetic reconstitution assay, which restored the proliferative loss in *BLVRB*-deficient T47D cells (Fig. 2H–K). Phenotypic correction was not evident using the redox-defective BLVRB^S111L^ mutant, confirming a requirement of BLVRB redox activity for tumor cells proliferation (Fig. 2H–K). These data establish that, independent of breast cancer subtype, BLVRB provides a necessary function in supporting cancer cell proliferation.

### BLVRB is a requisite component of breast cancer cell redox homeostasis

**Fig. 3:**
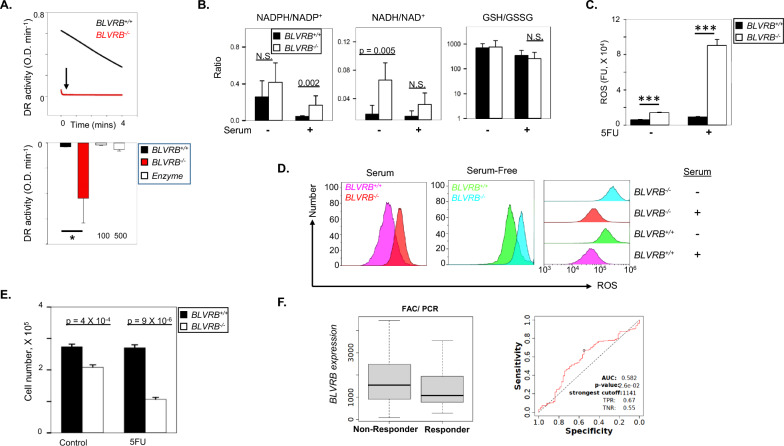
BLVRB maintains redox homeostasis and cytoprotection. **A** Cellular DCPIP reduction (DR) activity was quantified in SKBR3 *BLVRB*^+*/*+^ or *BLVRB*^*−/−*^ lysates (50 µg), demonstrating a striking and immediate reduction in *BLVRB*^*−/−*^ cells (*upper pane*l), with aggregated data (mean ± SEM, N = 2) shown in *lower panel*. Note that saturating recombinant BLVRB (a DCPIP substrate) at 100 and 500 nM fails to recapitulate cellular DR activity; *p*-value < 0.05 using unpaired t-test. **B** *BLVRB*^+*/*+^ or *BLVRB*^*−/−*^ cells were grown for 16 h in serum or serum-free (SF) conditions, followed by mass spectrophotometric quantification of NADPH/NADP^+^, NADH/NAD^+^, or GSH/GSSG ratios; data presented as the mean ± SEM (N = 5 or 6 samples); *p*-values were determined using unpaired t-test. **C** *BLVRB*^+*/*+^ or *BLVRB*^*−/−*^ cells were assayed for ROS accumulation by ROS-Glo assay, without (-) or with ( +) 5FU (5μM) for 24 h; data are presented as the mean ± SEM (N = 3); *p*-value determined by unpaired t-test, *** < 0.001. **D** Intracellular ROS accumulation was quantified using the cell-permeant fluorogenic probe CellROX Red, followed by flow cytometric detection of oxidized ROS (λ_ex/em_ 640/665 nm); shown are representative histograms for cells incubated in serum (*left panel*) or serum-free (*middle panel*) conditions, along with summary overlay (*right panel*), collectively demonstrating enhanced ROS in *BLVRB*^*−/−*^ cells that are exacerbated in serum-free conditions. **E** *BLVRB*^+*/*+^ or *BLVRB*^*−/−*^ cells were incubated without (control) or with 5 µM 5FU for 24 h, followed by cell number quantification (*left panel*); *p*-values determined by unpaired t-test. **F** *BLVRB*^*high*^ RNA expression predicts worse pathologic complete response (PCR) to FAC (5FU, doxorubicin, cyclophosphamide) in human breast cancers (all subtypes combined, *left panel*), with corresponding ROC (receiver operating curve) shown in the *right panel*

Given the clinical association of BLVRB^high^ with HER2^+^ subtype (Fig. 1J, K, and Supplementary Fig. 2), expanded studies were initially completed in HER2^+^ SKBR3-deficient BLVRB cells (referred to as *BLVRB*^*−/−*^) as an in vitro model. Interestingly, *BLVRB*^*−/−*^ demonstrated a pronounced shift towards a reduced intracellular redox state as evidenced by rapid (near-instantaneous) and complete DCPIP reduction compared to *BLVRB*^+*/*+^ cells (Fig. 3A). Enhanced reducing capacity was independent of BLVRB’s DCPIP catalytic activity [18], as demonstrated using saturating concentrations of exogenous BLVRB (up to 500 nM). To better elucidate the cause of reductive stress in *BLVRB*^*−/−*^ cells, we performed metabolic profiling by mass spectrometry under both metabolically active (serum-containing medium) and metabolic stress (serum-free) conditions. Consistent with an enhanced reductive cellular environment, *BLVRB*^*−/−*^ cells (compared to *BLVRB*^+*/*+^ cells) exhibited elevated NADPH/NADP^+^ (*p* = 0.002) and NADH/NAD^+^ ratios, the latter demonstrating statistical significance under serum-free conditions (*p* = 0.005, Fig. 3B). The unchanged glutathione (GSH/GSSG) redox ratio suggests that the observed reductive stress in BLVRB-deficient cells is specific to the NADH/NAD^+^ and NADPH/NADP^+^ redox couples and is independent of the glutathione system (Fig. 3B). This finding implies a distinct regulatory mechanism for maintaining redox balance in these cells, highlighting the divergence of NADPH/NADH and glutathione redox pathways in the context of BLVRB loss.

Disturbed redox homeostasis in *BLVRB*^*−/−*^ cells resulted in defective antioxidant handling with exaggerated ROS accumulation detected by both ROS-Glo assay (*p*-value < 0.001) and flow cytometric fluorescence (Fig. 3C, D); elevated baseline ROS accumulation in *BLVRB*^*−/−*^ cells was exaggerated in serum-free conditions (Fig. 3D). Exaggerated ROS accumulation was associated with loss of cytoprotection in *BLVRB*^*−/−*^ cells (Fig. 3E, *p* = 4 X 10^–4^). Furthermore, enhanced cytoprotective loss was evident using 5-fluorouracil (5FU) as an inducer of oxidative stress (Fig. 3E, *p* = 9 X 10^–6^), associated with exaggerated ROS accumulation (Fig. 3C, *p* < 0.001)[62, 63] and cytotoxicity (Fig. 3E). In agreement with these findings, analysis of chemotherapy response using breast cancer datasets [57] demonstrated a positive correlation between *BLVRB*^*high*^ cohorts and resistance to the 5FU-based therapeutic regimen FAC (5FU, doxorubicin, and cyclophosphamide, Fig. 3F).

### BLVRB regulates reprogramming of the metabolic network 

**Fig. 4:**
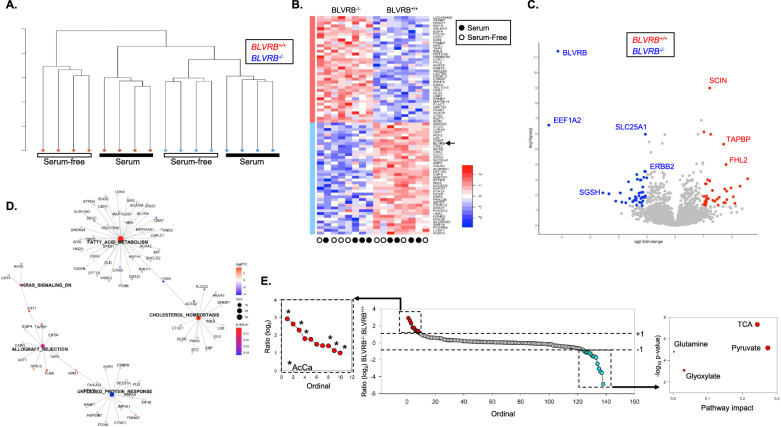
Integrated proteomic and metabolic analyses identify dysregulated adaptive pathways in *BLVRB*-deficient cells. **A** Unsupervised hierarchical clustering dendrogram of proteomic data (N = 2, 553 proteins) demonstrates phenotypic segregation based on both genotype (*BLVRB*^*−/−*^* vs. BLVRB*^+*/*+^) and experimental condition (± serum). **B** The protein expression heatmap demonstrates changes both by genotype (*BLVRB*^*−/−*^* vs. BLVRB*^+*/*+^) and experimental condition (± serum); the scale bar is shown. **C** Volcano plot delineates differentially expressed proteins displaying greatest statistical significance in *BLVRB*^*−/−*^ (blue, downregulated) *vs. BLVRB*^+*/*+^ (red, upregulated) cells. **D** Network plot depicts significantly enriched *BLVRB*^*−/−*^ pathways identified by MSigDB during stress. Major nodes denote pathways, and the numbers of enriched proteins contained within a pathway are represented by node size; statistical significance (*p*.adjust) is represented by a scaled color (red: more significant; blue: less significant). Smaller nodes surrounding a pathway denote pathway-enriched proteins, and scaled color represents log_2_ fold-change (log_2_ FC) (red: upregulated in *BLVRB*^*−/−*^ cells; purple: downregulated in *BLVRB*^*−/−*^ cells). Edges between pathway nodes and protein nodes define proteins belonging to pathways, and select DE-identified proteins are labeled. Note the identification of the unfolded protein response (UPR, upregulated) and lipid metabolic pathways (cholesterol and fatty acid metabolism, downregulated) as key adaptive perturbations. **E** *BLVRB*^+*/*+^ and *BLVRB*^*−/−*^ cells (N = 6/genotype) were grown in serum-free conditions for 16 h, followed by metabolomic quantification by mass spectrometry. The ordinate plot (*center*) shows the log_2_ ratio (*BLVRB*^*−/−*^:*BLVRB*^+*/*+^) of N = 138 quantifiable metabolites; the inset (*left*) delineates the metabolite subset displaying log_2_ fold-change ratio ≥  + 1, *p*-value ≤ 0.01 (N = 10, enriched in acetyl carnitines (6 of 10 metabolites), red), while the inset (*right*) displays the pathway enrichment analysis of the metabolite subset displaying log2 ratio ≤ -1, *p*-value ≤ 0.01 (N = 12 enriched in TCA cycle metabolites, turquoise); the enrichment plot was generated using MetaboAnalyst, *Version* 6.0 [51]

We completed the proteomic analysis of *BLVRB*^+*/*+^ and *BLVRB*^*−/−*^ cells under both basal (with serum) and metabolic stress (serum-free) conditions (N = 4 samples/genotype/condition) to better dissect BLVRB-mediated pathways regulating redox homeostasis in breast cancer metabolic responses [64]. Unsupervised hierarchical clustering of normalized mass spectral data revealed clear phenotypic segregation both by genotype (*BLVRB*^+*/*+^ vs. *BLVRB*^*−/−*^) and experimental condition, demonstrating dynamic proteomic adaptations specifically delimited by BLVRB expression and/or the presence of metabolic stress (Fig. 4A). Aggregated data analysis identified a limited number of differentially expressed proteins (N = 288, adjusted *p*-value < 0.05), of which 38 were up- and 40 were down-regulated (-1 ≤ log2FC ≥ 1, Fig. 4 B, C). Expectedly, BLVRB exhibited the most pronounced decrease along with the translational regulator EEF1A2 (eukaryotic translation elongation factor 1 alpha 2) and the lysosomal sulfamidase (SGSH); the receptor tyrosine kinase HER2 (ErbB2) was also down-regulated (Fig. 4C). Among the most significantly abundant proteins detected in *BLVRB*^+*/*+^ vs. *BLVRB*^*−/−*^ cells were those involved in cytoskeletal and membrane maintenance including SCIN (scinderin, an actin-capping protein), COTL1 (an F-actin binding protein), FHL2, and TAPBP (both associated with membrane assembly and ER membrane maintenance).

We generated interactive networks using highly-curated Hallmark data sets to delineate globally-affected pathway perturbations, and identified the unfolded protein response (UPR, upregulated) and lipid metabolic (cholesterol and fatty acid metabolism) pathways (downregulated) as the primary stress-responsive networks in BLVRB deficient cells (Fig. 4D). The fatty acid and cholesterol metabolic pathways are interconnected through fatty acid synthase (FASN, Fig. 4D), an enzyme responsible for catalyzing de novo biosynthesis of long-chain saturated fatty acids from acetyl-CoA and malonyl-CoA, utilizing NADPH as a cofactor [65]. Quantification of secreted metabolites by mass spectrometry under serum starvation confirmed selective excretion of acylcarnitines in *BLVRB*^*−/−*^ cells (compared to *BLVRB*^+*/*+^), corroborating the observed perturbations in fatty acid/cholesterol metabolic networks (Fig. 4E and Supplementary Table 2). Conversely, *BLVRB*^*−/−*^ cells exhibit a decrease in the secretion of TCA cycle metabolites (succinate, citrate, pyruvate, malate, fumarate, Supplementary Table 1), suggesting selective preservation of functional intracellular TCA (and pyruvate) metabolic networks required for acetyl-CoA oxidation (Fig. 4E). Consistent with these findings, BLVRB loss results in the downregulation of SLC25A1 (Fig. 4C), a key transporter for mitochondrial export of citrate, a vital precursor for fatty acid synthesis [66]. Collectively, integrated proteomic and metabolomic analyses identified significant metabolic dysregulation following BLVRB loss, characterized by disordered TCA and fatty acid metabolic networks.

### BLVRB deficiency triggers the unfolded protein response and endoplasmic reticulum stress

**Fig. 5:**
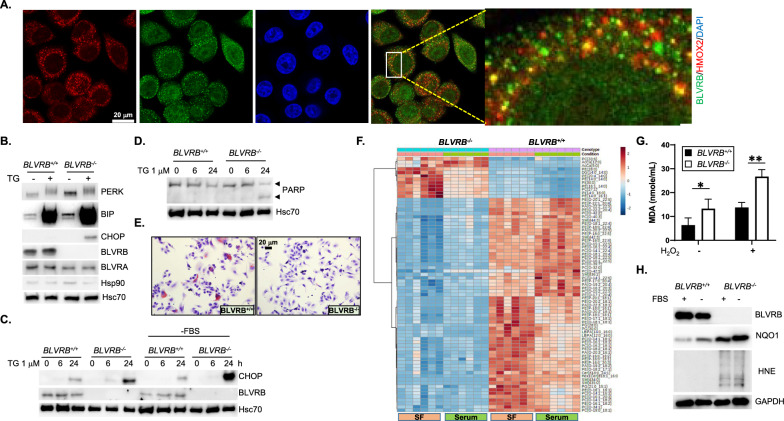
BLVRB loss induces the unfolded protein response (UPR) and ER stress. **A** Confocal immunofluorescence of SKBR3/*BLVRB*^+*/*+^ cells demonstrates co-localization of BLVRB (green) with the ER-resident protein HMOX2 (red), with nuclei stained with DAPI (blue); size marker (20 µm) is shown. **B–D** Cells (*BLVRB*^+*/*+^ and *BLVRB*^*−/−*^) were incubated with 1 µM thapsigargin (TG) for 16 h (*Panels B, D*), or for time course (0, 6, 24 h, *Panel C*), followed by immunoblot analysis (20 µg/lane) for makers of ER stress. Consistent with increased sensitivity to ER stress, *BLVRB*^*−/−*^ cells exhibit elevated levels of PERK, BiP, and CHOP which is exaggerated by thapsigargin treatment (*Panel B*), or PARP cleavage (*Panel D*), along with the time-dependent increase in CHOP that is enhanced by serum starvation (-FBS, fetal bovine serum) as stressor (*Panel C*); for all *Panels*, Hsc70 serves as loading control. **E** *BLVRB*^*−/−*^ cells stained with Oil Red O staining (Red) show a significant decrease in cellular lipid content compared to *BLVRB*^+*/*+^ cells; size marker (20 µm) is shown. **F** Cells (*BLVRB*^+*/*+^ and *BLVRB*^*−/−*^, N = 6/genotype/condition) were grown for 16 h in serum or serum-free (SF) conditions, followed by targeted (N = 495 member) mass spectrometric lipidomic analyses; the heat map shows selective decrease in phospholipids (phosphatidylserine (PS) and phosphatidylethanolamine, PE) in *BLVRB*^*−/−*^ compared to *BLVRB*^+*/*+^ cells. Scale bar is shown. (**G**) Cells (*BLVRB*^+*/*+^ and *BLVRB*^*−/−*^) were incubated with 25 µM H_2_O_2_ (16 h), followed by MDA (malonaldehyde) quantification by ELISA; data are presented as mean ± SEM (N = 3 replicates), *p*-value * < 0.05, ** < 0.01 by unpaired t-test. **H** *BLVRB*^+*/*+^ and *BLVRB*^*−/−*^ cells were grown for 16 h without (-FBS) or with (+ FBS, fetal bovine serum), followed by immunoblot analysis (20 µg/lane) for detection of the lipid peroxidation product HNE (4-hydroxynonenal), or the superoxide scavenger NQO1

The endoplasmic reticulum (ER) maintains proper protein folding and lipid metabolism and is disrupted by reductive stress, which interferes with disulfide bond formation [67, 68], thereby providing a mechanism whereby BLVRB deficiency could trigger the unfolded protein response (UPR), ER stress, and lipid metabolic perturbations. ER stress is mediated by three ER receptors (IRE1 [inositol-requiring enzyme 1], PERK [PKR-like ER kinase], and ATF6a [activating transcription factor 6]), collectively bound to the ER chaperone protein glucose-regulated protein 78 (GRP78; BiP), and displaced (upon ER stress) with concomitant release of thiol isomerases that catalyze protein folding [69]. Confocal microscopy confirmed partial co-localization of BLVRB with the ER-resident protein HMOX2 [70], suggesting a spatially-restricted redox function integrated with heme processing within this organelle (Fig. 5A). Indeed, *BLVRB*^*−/−*^ cells displayed signs of persistent UPR and ER stress under basal conditions as demonstrated by elevated levels of PERK and BiP, patterns that were further amplified using the ER stress inducer thapsigargin or serum starvation (Fig. 5B, C). Notably, the cytosolic chaperone Hsp90 that senses the accumulation of cytosolic unfolded proteins remained unchanged, suggesting spatially restricted proteotoxic stress (Fig. 5B).

Downstream effect(s) of an activated UPR in *BLVRB*^*−/−*^ cells include repression of protein translation as a compensatory mechanism to mitigate against the accumulation of unfolded proteins [69], results predicted by the proteomic studies demonstrating downregulation of the translation initiator EEF1A2 (Fig. 4C). Indeed, BLVRB-deficient cells exhibited time-dependent increase in the downstream PERK effector CHOP (C/EBP homologous protein), a pro-apoptotic transcription factor [71]). CHOP induction in *BLVRB*^*−/−*^ cells was augmented by serum starvation (Fig. 5C), sensitizing cells to apoptosis as evidenced by PARP cleavage (Fig. 5D). Collectively, these findings demonstrate a requisite and likely spatially-restricted function of BLVRB in maintaining ER proteostasis, exacerbated with metabolic stress.

### BLVRB deficiency impairs lipid metabolism and alters membrane functionality

The known capacity of the ER in regulating lipid synthesis [72], coupled with the identification of a perturbed fatty acid metabolic pathway (Fig. 4D), prompted further investigations into associated lipid defects. Loss of BLVRB leads to a marked reduction in cellular lipid content, as evidenced by Oil Red O staining, a quantifiable marker of neutral lipid accumulation (Fig. 5E). To better characterize quantitative and qualitative differences in lipid composition, we completed lipidomic profiling under both normal and serum-free conditions (N = 6/genotype/condition). Differential lipidomic analyses across the variables (genotype/condition) identified selective depletion of phospholipids in *BLVRB*^*−/−*^ cells, particularly phosphatidylserine (PS) and phosphatidylethanolamine (PE) (Fig. 5F). These phospholipids are essential components of cellular membranes, forming the lipid bilayer structures comprising subcellular organelles and cell membranes. Indeed, the proteome data reinforce the lipidomic analyses, given the significant changes in the expression of several proteins crucial for membrane and cytoskeletal maintenance (SCIN, COTL1, FHL2, TAPBP) upon BLVRB loss (Fig. 4C).

We extended these observations to address additional lipid-associated defects mediated by BLVRB depletion. Consistent with elevated ROS levels (Fig. 3C, D), BLVRB-deficient cells demonstrated exaggerated lipid peroxidation, evidenced by increased omega-6 fatty acid adducts of malondialdehyde (MDA) (Fig. 5G) and 4-hydroxynonenal (4-HNE) (Fig. 5H). Lipid peroxidation defect(s) were further aggravated by stress induced by H₂O₂ or serum starvation (Fig. 5G, H). Indeed, the superoxide scavenger NQO1 (NAD(P)H dehydrogenase [quinone]), a well-established NRF2 target [73] was upregulated in BLVRB-deficient cells, and exaggerated in serum-free stress conditions (Fig. 5H), although insufficient to fully compensate for the lipid peroxidation damage. These results suggest that BLVRB contributes to membrane functionality through dual mechanisms: maintenance of phospholipid homeostasis and as a spatially active cytoprotectant against membrane lipid peroxidation damage.

### Plasma membrane dysfunction reduces the membrane localization of HER2 and transferrin receptors.

**Fig. 6:**
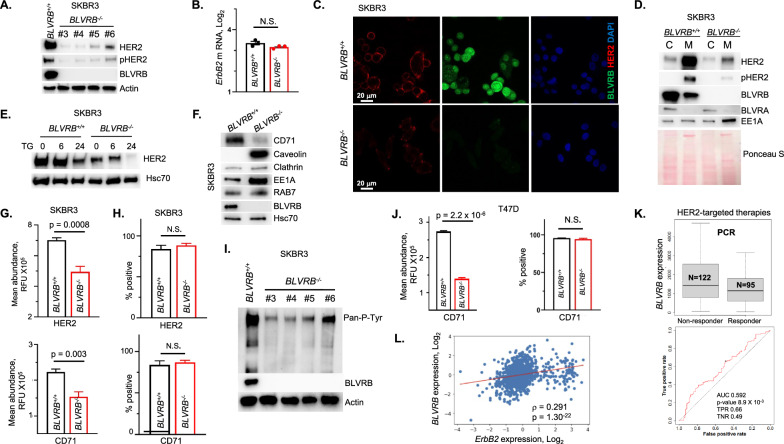
BLVRB loss reduces the membrane localization of HER2 and transferrin receptors. **A** Immunoblot (20 µg/lane) demonstrates loss of HER2 protein and its phosphorylation (Y1221/Y1222) in all *BLVRB*^*−/−*^ clones; Actin is shown as a loading control. **B** *ErbB2* Q-PCR of *BLVRB*^+*/*+^ or *BLVRB*^*−/−*^ cells is presented as mean ± SEM normalized to actin (*Actb*, N = 3/cohort); *p*-value (not significant, N.S.) using unpaired t-test. **C** Confocal microscopy demonstrates loss of total and cell-surface HER2 in *BLVRB*^*−/−*^ compared to *BLVRB*^+*/*+^ cells; size marker (20 µm) is shown. **D** Subcellular fractionation demonstrate enriched membrane HER2, with evidence for both cytoplasmic (predominant) and membrane-bound BLVRB in *BLVRB*^+*/*+^ cells; note the clear loss of total and membrane-bound HER2 in *BLVRB*^*−/−*^ cells occurring in conjunction with EE1A membrane translocation (20 µg lysates/lane). Ponceau S protein staining as a loading control. **E** ER stress induced by 1 µM thapsigargin (TG) leads to exaggerated time-dependent HER2 loss in *BLVRB*^*−/−*^ compared to BLVRB^+/+^ cells (20 µg lysates/lane); Hsc70 is used as the loading control. **F** Immunoblot (20 µg/lane) demonstrates downregulation of transferrin receptor (CD71) in *BLVRB*^*−/−*^ compared to BLVRB^+/+^ cells, with concomitant increase in caveolin and EE1A, but not RAB7 and clathrin. **G**, **H** Flow cytometric analyses of *BLVRB*^+*/*+^ and *BLVRB*^*−/−*^ cells (fixed, non-permeabilized) show cell-surface quantification of HER2 and CD71 abundance (mean fluorescence intensity (MFI), *Panel G*) or percent positivity (*Panel H*); data are presented as mean ± SEM from N = 5—6 technical and biologic replicates; *p*-values are shown; N.S. not significant. **I** Pan-phosphotyrosine antibody immunoblot reveals a global decrease in RTK activity across all BLVRB knockout clones. **J** Flow cytometric analyses of T47D/*BLVRB*^+*/*+^ and T47D/*BLVRB*^*−/−*^ cells (fixed, non-permeabilized) show cell-surface quantification of CD71 (TfR) abundance (mean fluorescence intensity (MFI), *left panel*) or percent positivity (*right panel*); data are the mean ± SEM from N = 3 technical and biologic replicates; *p*-values are shown; N.S. not significant. **K** Box plots (displaying the median, upper quartile, minimum, and maximum values, *upper panel*) and corresponding ROC (receiver operator characteristic) curve (*lower panel*), demonstrate that elevated *BLVRB* expression in human breast cancer is associated with a worse pathological complete response (pCR) to HER2 targeted (Lapatinib and Trastuzumab) therapies; graphs and statistics were generated in silico [57]. (L) Spearman’s rank correlation analysis of *ErbB2* and *BLVRB* mRNA expression in breast cancer samples from the TCGA cohort (N = 1082, all subtypes) show a statistically significant positive correlation between *ErbB2* and *BLVRB* expression (ρ = 0.291, *p* = 1.30 × 10^−22^)

BLVRB-regulated membrane function led us to hypothesize that HER2 stability and/or trafficking would explain prognostic associations in HER2^+^ breast cancer cohorts (vide supra). Indeed, we observed concurrent downregulation of HER2 protein (consistent with proteomic studies in Fig. 4) and a proportional to total HER2 abundance decrease in HER2 activation (Y1221/Y1222 phosphorylation) in all *BLVRB*^*−/−*^ clones (Fig. 6A). Notably, this downregulation occurred at the protein level without any significant changes in *ErbB2* mRNA (Fig. 6B), consistent with a post-transcriptional regulatory mechanism. Confocal microscopy and subcellular fractionation studies demonstrated selective enrichment of HER2 in membrane fractions, with proportional loss of both cytoplasmic and membrane-associated HER2 (and phosphorylated HER2) in *BLVRB*^*−/−*^ cells (Fig. 6C, D). Subcellular fractionation confirmed that BLVRB was also distributed in both membrane and cytosolic fractions in *BLVRB*^+*/*+^ cells, although most prominent in the cytoplasm. Induction of ER stress with thapsigargin caused exaggerated time-dependent HER2 loss in *BLVRB*^*−/−*^ cells (Fig. 6E), consistent with previous report implicating ER stress in a HER2 degradation mediated by mTOR signaling [74].

Endosomal recycling of HER2 plays an important role in regulating its oncogenic signaling [75], and loss of membrane-associated HER2 in *BLVRB*^*−/−*^ cells developed in conjunction with membrane accumulation of the early endosomal marker antigen 1 (EEA1) (Fig. 6D), but not the late endosomal marker RAB7 (Fig. 6F). EEA1 is essential for tethering and fusion between early endosomes and other vesicles, suggesting a mechanism for potential disruption in endosome maturation and/or cargo sorting/trafficking [76] (vide infra). Indeed, the increase in caveolin (but not clathrin) in *BLVRB*^*−/−*^ cells specifically suggested restricted defect(s) involving caveolin-mediated endocytosis (Fig. 6F), which is known to regulate HER2 cellular trafficking [77]. Interestingly, a more generalized impairment in endosomal recycling upon BLVRB loss was confirmed by observing similar effects on the transferrin receptor (TfR, CD71), a well-established prototypic model for endocytic recycling critical for iron metabolism [78]. Similar to diminished cell-surface HER2 expression in *BLVRB*^*−/−*^ cells, transferrin receptor antigenic loss was also evident by immunoblot (Fig. 6F) and confirmed by flow cytometry, which demonstrated cell-surface loss of both receptors (HER2 and CD71) in SKBR3/*BLVRB*^*−/−*^ cells without affecting cellular percent positivity (Fig. 6 G, H). Analogous patterns were evident for receptor tyrosine kinases (RTKs), where pan-phosphotyrosine antibody staining revealed a global decrease in RTK activity across all SKBR3 *BLVRB* knockout clones (Fig. 6I), further confirming global membrane dysfunction in *BLVRB*^*−/−*^ cells. To determine if comparable cell-surface defect(s) were evident in a second BLVRB-expressing cell line, we extended these studies by characterizing CD71 expression in previously characterized T47D (HER2-, Luminal A) cells (Fig. 2G, vide supra). Similar to results in SKBR3/*BLVRB*^*−/−*^ cells, T47D/*BLVRB*^*−/−*^ demonstrated statistically-significant CD71 loss (*p* = 2.2 × 10^–6^) compared to parental T47D/*BLVRB*^+*/*+^ cells (Fig. 6J), with no effect on CD71 percent distribution (mirroring effects in SKBR3/*BLVRB*^*−/−*^). Previous studies demonstrated that dysregulated HER2 endosomal recycling provides a mechanism whereby cancer cells develop resistance to HER2-targeted therapies by allowing HER2 to evade drug actions while maintaining signaling activity [75, 79]. Indeed, our analysis using clinically available datasets [57] identified pCR (pathological complete response) that indicates resistance to HER2-targeted therapies (lapatinib and trastuzumab) in *BLVRB*^*high*^-expressing tumors (Fig. 6K), highlighting the importance of BLVRB-mediated endosomal trafficking of HER2 as a putative contributor of resistance to HER2-targeted therapies and poor survival of patients with *BLVRB*^*high*^ tumors (Fig. 1J, K).

Finally, we used human and murine databases to further define *ErbB2*/*BLVRB* contextual relationships. Correlation analysis using TCGA database (N = 1,082) across all breast cancer subtypes revealed a highly significant association between *ErbB2* and *BLVRB* expression (ρ = 0.291, *p* = 1.30 × 10^−22^, Fig. 6L). Similarly, analysis of scRNA data sets [80] of mammary tissue from MMTV/*ErbB2* mice (n = 4; 12 weeks age) revealed a strong correlation between *Blvrb* and *ErbB2* expression. Developmental kinetics of mammary tumorigenesis in this mouse model suggest that the mammary tissue of 12-week-old mice is comprised of (i) normal MECs, (ii) DCIS lesions, and (iii) microscopic low-grade tumors with a gradual increase of ErbB2 expression as lesions progress [81]. In contrast to cancer cells, MECs display very low *ErbB2* and undetectable *Blvrb* (Fig. 1D–F, vide supra), and we confirmed a strong correlation between *Blvrb* and *ErbB2* in epithelial cells (ρ = 0.56, *p* < 1.74 X10^−9^), synchronous with tumor progression (Supplementary Fig. 3B, C).

### Silencing BLVRB suppresses mammary tumorigenesis in vivo.

**Fig. 7:**
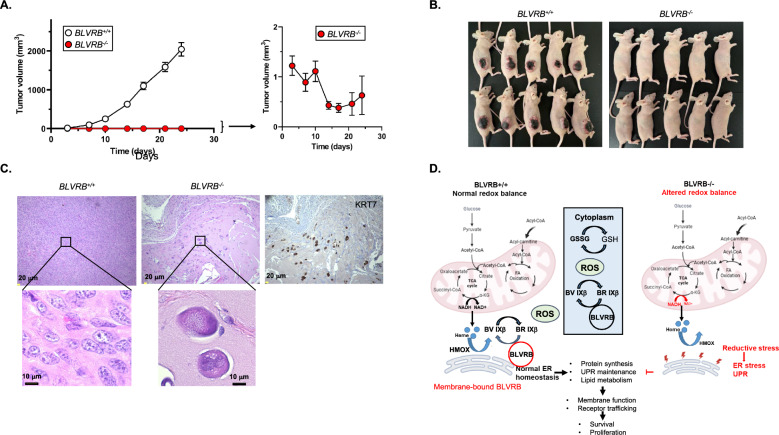
BLVRB silencing suppresses mammary tumorigenesis in vivo. **A**, **B** Tumor growth kinetics of *BLVRB*^+*/*+^ and *BLVRB*^*−/−*^ cells were established by subcutaneous flank implantations (2 × 10^6^ cells/site) in female athymic nude mice, data presented as the mean. ± SEM (N = 10 mice/cohort); the inset (*Panel A*) shows re-scaled *BLVRB*^*−/−*^ growth curves, with photographs (Day 24 post-implantation) provided in *Panel B*. **C** Histological sections (Day 24) stained with hematoxylin and eosin (H&E) show atypical cells with high nuclear: cytoplasm ratio, moderate nuclear pleomorphism, coarse chromatin, prominent nucleoli and brisk mitotic activity in *BLVRB*^+*/*+^ mice tumors, contrasting with enlarged, mummified-appearing, highly atypical single cells with foamy cytoplasm scattered in a fibrous background in *BLVRB*^*−/−*^ mice tumors. The positive immunohistochemistry staining for cytokeratin 7 (KRT7) in *BLVRB*^*−/−*^ tumors establishes the epithelial origin of these highly atypical cells. **D** The summary schema depicts a model for BLVRB function(s) in breast cancer. Heme biosynthesis occurs from the condensation of TCA cycle-derived succinyl-CoA and glycine, and cytotoxic free heme is sequentially degraded by HMOXs to generate biliverdins (BVs). In BLVRB-expressing breast cancer cells (*left panel*), BLVRB is upregulated and spatially distributed in both cytoplasm and endoplasmic reticulum membranes, where its cytoprotective effect(s) is maintained by a BV IXβ to BR IXβ redox cycle that regenerates the BR and maintains redox homeostasis. The cytoplasmic GSH/GSSG couple functions as the predominant antioxidant for cytoplasmic components, although membrane-bound BLVRB (red circle) as proposed provides the critical membrane-protective effects mediated by lipophilic BR IXβ protection against lipid peroxidation. With BLVRB loss (*right panel*), altered redox balance and defective ER membrane-protective functions result in ER proteotoxic stress and associated defects in UPR and phospholipid synthesis; membrane dysfunction, a consequence of combined lipid peroxidation and altered phospholipid composition, leads to defective trafficking of membrane proteins including HER2 and transferrin receptors

We confirmed the consequences of BLVRB deficiency in vivo using SKBR3 tumor xenografts of *BLVRB*^+*/*+^ or *BLVRB*^*−/−*^ cells subcutaneously implanted in the flanks of female athymic nude mice (N = 10 mice/group). All *BLVRB*^+*/*+^ implants developed tumors, exhibiting rapid growth over the study period (Fig. 7A). In contrast, *BLVRB*^*−/−*^ implants showed highly-retarded tumor growth, with a mean tumor volume of less than 10 mm^3^ at all time points (Fig. 7B, *p* < 0.001). *BLVRB*^*−/−*^ implants were largely quiescent and displayed no growth over the initial 10-day period, with progressive tumor regression after Day 10 post-injection. Histopathology of a single dissected tumor remnant (Day 24) confirmed the presence of necrotic, highly atypical cells with foamy cytoplasm scattered in a fibrous background in *BLVRB*^*−/−*^ mice tumors. These results sharply contrasted with the typical characteristics of malignant cells evident in *BLVRB*^+*/*+^ tumors (Fig. 7C).

## Discussion

Our collective data identify and validate BLVRB as a unique and novel cellular target in breast cancer pathogenesis and progression. The conclusions are predicated on both cell biology and clinically relevant data sets and are best characterized for HER2-positive breast cancer, which demonstrates the most abundant BLVRB expression using confirmatory in vitro models. Based on our studies, a model is proposed whereby BLVRB loss induced chronic reductive stress orchestrates pleiotropic cellular effects resulting in dysregulated ER proteostasis and lipid metabolic defects, with secondary effects on plasma membrane function (Fig. 7D *schema*). A generalizable membrane function defect manifests as the simultaneous loss of cell-surface HER2 and CD71 receptors, accompanied by global effects on RTK activity. Validation is established in a second BLVRB-expressing cell line T47D (Luminal A), which also demonstrates a significant loss of CD71 in *BLVRB*-knockout cells. Coordinate involvement of the HER2 endosomal recycling pathway is implicated based on EE1A and caveolin accumulation, presumably by modulating the stability of caveolin-enriched lipid rafts that may affect HER2-targeted therapies [75, 77, 82]. Interestingly, CD71 is frequently overexpressed in breast cancer with the gradual increase in expression from DCIS to invasive ductal carcinoma [83] and is also considered an attractive target for directed therapy [84]. Xenograft transplantation studies demonstrate striking loss of *BLVRB*^−/−^ engraftment, consistent with a global membrane defect and exaggerated cellular effect(s) extending beyond HER2 (and/or CD71 receptor) functions. Indeed, our a priori model suggests that HER2 disruption is a secondary event downstream of BLVRB-associated membrane dysfunction and that BLVRB-regulated redox homeostasis and cytoprotection may have effects in expanded breast cancer subtypes displaying abundant BLVRB expression.

In addition to phenotypic effects regulating membrane homeostasis and cell-surface receptor expression, our proteomic-based network analysis identified dysregulated pathways involving both the unfolded protein response and fatty acid/cholesterol metabolism as primary adaptive perturbations in BLVRB-deficient cells. Defects in both pathways were validated in cellular and metabolic profiling studies; a potential limitation of the metabolomic profiling is use of enzymatic detachment (trypsinization) which may result in metabolite leakage, although excellent concordance between methods (trypsinization and scraping) has recently been reported [85]. Heme synthesis occurs primarily in the mitochondria and cytoplasm, although the accumulation of intracellular free heme contributes to chronic reductive stress through its association with ER-anchored HMOX. Unlike oxidative stress, reductive stress is associated with excessive accumulation of reducing equivalents (in our case, NADH and NADPH couples, not involving the glutathione GSSG/GSH system) that lead to ROS accumulation, lipid peroxidation, and ER disturbances [reviewed, [64]. Independence from the GSSG/GSH system is explained by spatially-restricted membrane cytoprotection independent of cytoplasmic glutathione. Indeed, cell fractionation studies confirm that BLVRB in breast cancer cells is spatially distributed both in membranes and cytoplasm – and co-localizes with HMOX in the ER membrane – providing pleiotropic and requisite effects in maintaining cell-surface (and ER) membrane functionality. Bilirubin is lipophilic and has been proposed to retain a membrane-specific antioxidant function protecting against lipid peroxidation [10], similar to the results evident in our studies. Although intracellular BR levels are low (~ 20 – 50 nM) compared to the more abundant (millimolar) glutathione (GSSG/GSH) couples, cellular ROS generated at the membrane provides a plausible mechanism for an amplifiable BV to BR redox cycle retaining spatially-restricted membrane cytoprotection [10].

BLVRB redox and cytoprotective functions are exaggerated in the presence of stressors, readily evident in the stress-delimited adaptive changes in both the BLVRB^−/−^ proteome and lipidome. A comparable stress-associated cancer phenotype was evident in BLVRB-deficient SKBR3 cells, which displayed exaggerated proliferative loss and lipid peroxidation defect(s) using either 5FU or serum starvation as stressors or thapsigargin as an ER-restricted stressor. In actively proliferating cells, the ER maintains the oxidizing environment required for the formation of disulfide bonds and proper protein folding, whereas reductive stress interferes with normal disulfide bond formation, resulting in activation of UPR and ER stress [67]. Prolonged UPR activation inhibits translation (EEF1A2 downregulation, Fig. 4C), phospholipid synthesis (Fig. 5F) [86], and sensitizes cells to apoptosis (Fig. 5D) and cell death [67, 68], providing a coordinated model for downstream cellular effects. Similarly, NADPH is a crucial electron donor for several reductive synthetic reactions in cancer [87] and provides the reducing equivalents for fatty acid synthase (FASN, identified in our network plot). FASN is the rate-limiting enzyme for fatty acid synthesis from acetyl-CoA [88] and provides an NADPH-dependent pathway linked to the dysregulated membrane composition identified by lipid profiling. Finally, previous data in BLVRB-deficient induced pluripotent stem cells (iPSCs) identified a glutamine-restricted defect in TCA entry, coupled with a requisite BLVRB function in providing support for the pentose phosphate pathway (PPP), which is the primary source of cellular NADPH generation critical for cellular reductive functions [18]. Interestingly, we also observed a significant decrease in the secretion of TCA cycle metabolites in *BLVRB*^*−/−*^ cells (Fig. 4E), suggesting adaptive mechanisms to preserve functional intracellular TCA metabolites.

BLVRB retains various characteristics that make it an attractive cellular target for novel cancer-directed therapeutics. Murine *Blvrb* is dispensable for organ and cellular development, and *Blvrb*-deficient mice display no organ pathology over 2 years of follow-up, although a stress-restricted hematopoietic phenotype is evident when injected with 5-fluorouracil (5FU) as a stressor [41]. In humans, erythrocytes express BLVRB as a non-physiological methemoglobin reductase [37], although it functions as the target redox coupler in cytochrome b5 reductase (*CYB5R3*)-deficient patients with methemoglobinemia. BLVRB is differentially expressed in malignant mammary cells compared to normal counterparts (Figs. 1D, E, 2A), and *BLVRB* expression is greater than *BLVRA* (or *HMOX1/HMOX2*) across the majority (53/60) of the NCI60 cell lines. Importantly, BLVRA fails to rescue the phenotype induced by BLVRB silencing. The non-redundancy of BLVRA and BLVRB is not unexpected given their clear substrate specificities. BLVRA utilizes BV IXα as the sole substrate, although BLVRB is promiscuous and catalyzes the NAD(P)H-dependent reduction of a variety of flavins (flavin mononucleotide (FMN), flavin adenine dinucleotide (FAD), riboflavin), methemoglobin (MetHb^[+3]^) and ferric^[+3]^ iron, pyrroloquinoline quinone, and isomer-specific BVs (IXβ, IXγ, IXδ)–BRs (IXβ, IXγ, IXδ) [89, 90]. Although partially homologous to BLVRB, BLVRA possesses a DNA-binding domain, a nuclear localization sequence, and a nuclear export sequence that are absent in BLVRB [89, 91]. Structural differences, restricted substrate specificities, and differential tissue distribution with prognostic associations highlight their diverse, non-overlapping functions in cancer in general, and breast cancer as outlined in our studies. Interestingly, recent work has demonstrated a putative function of BLVRB as an S-nitroso-CoA-dependent nitrosyltransferase that facilitates the transfer of nitric oxide (NO) to specific cysteine residues within target proteins, including HMOX2, insulin receptor (INSR), and insulin receptor substrate 1 (IRS1), ultimately modulating their function [92]. Whether a comparable BLVRB function exists for the modulation of breast cancer proteins and/or receptors during quiescence or stress remains unestablished.

Human BLVRB contains a single dinucleotide-binding domain (Rossman fold) that accommodates both NAD(P)H (or NADH) and substrate(s) within the verdin/flavin binding pocket; a compulsory ordered kinetic mechanism has been proposed in which NAD(P)H binding followed by substrate (*i.e.* BV) results in the sequential release of product (i.e. BR) and oxidized cofactor [93, 94]. Formulated on x-ray crystallography [38] and thermodynamic modeling [95], we previously identified BLVRB inhibitors displaying inhibitory constants (*K*_i_) in the low micromolar [38] range, with no evidence to date for structural or preferred partitioning for substrate(s) or inhibitors outside of the BLVRB binding pocket. A subsequent computational screen using an expanded set of BLVRB/inhibitor trimolecular complexes yielded an expanded library of de novo synthesized small molecule inhibitors retaining excellent pharmacokinetic and metabolic characteristics, oral bioavailability, and no off-target effects [96]. These redox inhibitors may now be used to address target validation in relevant *BLVRB*^*high*^ breast cancer model systems, using well-characterized readouts relevant to cellular reductive capacity, ER stress, and cell-surface receptor HER2 processing; based on current models, expanded indications beyond HER2^+^ breast cancer are also predicted.

## Conclusions

Here, for the first time, we identified BLVRB as a novel requisite metabolic regulator in breast cancer, where it maintains redox homeostasis, endoplasmic reticulum proteostasis, proper lipid composition, and plasma membrane functionality, impacting the function of multiple oncogenic receptors such as HER2 and transferrin receptor (CD71). Together, our mechanistic studies establish BLVRB as a novel therapeutic target with potential translation for breast cancer therapy. Our recent progress in the identification of a novel small molecule BLVRB inhibitor that retains the capacity for targeted substrate inhibition of the BLVRB active site [96] opens a new prospect in the development of new targeted therapy for breast cancer with high expression of BLVRB. In contrast to antibody-based receptor-targeted therapies or receptor tyrosine kinase inhibitors, which target specific receptors, BLVRB inhibitors offer a distinct mechanism of action that may complement and enhance conventional therapies. Future studies are required to validate whether BLVRB inhibitors recapitulate the established effects of the genetic depletion of BLVRB in breast cancer.

## Supplementary Information

Supplementary material 1.

Supplementary material 2.

Supplementary material 3.

Supplementary material 4.

Supplementary material 5.

Supplementary material 6.

## Data Availability

The datasets used and/or analyzed during the current study are available from the corresponding author upon reasonable request.
