# Supplementary material for: Biliverdin reductase B as a new target in breast cancer
Source: Breast Cancer Res. 2025 Oct 16;27:179. doi: 10.1186/s13058-025-02147-x (PMC12532840; doi:10.1186/s13058-025-02147-x)
Supplement: Supplementary file 1 — Supplementary material 1. [file 13058_2025_2147_MOESM1_ESM.pptx]

## Slide 1
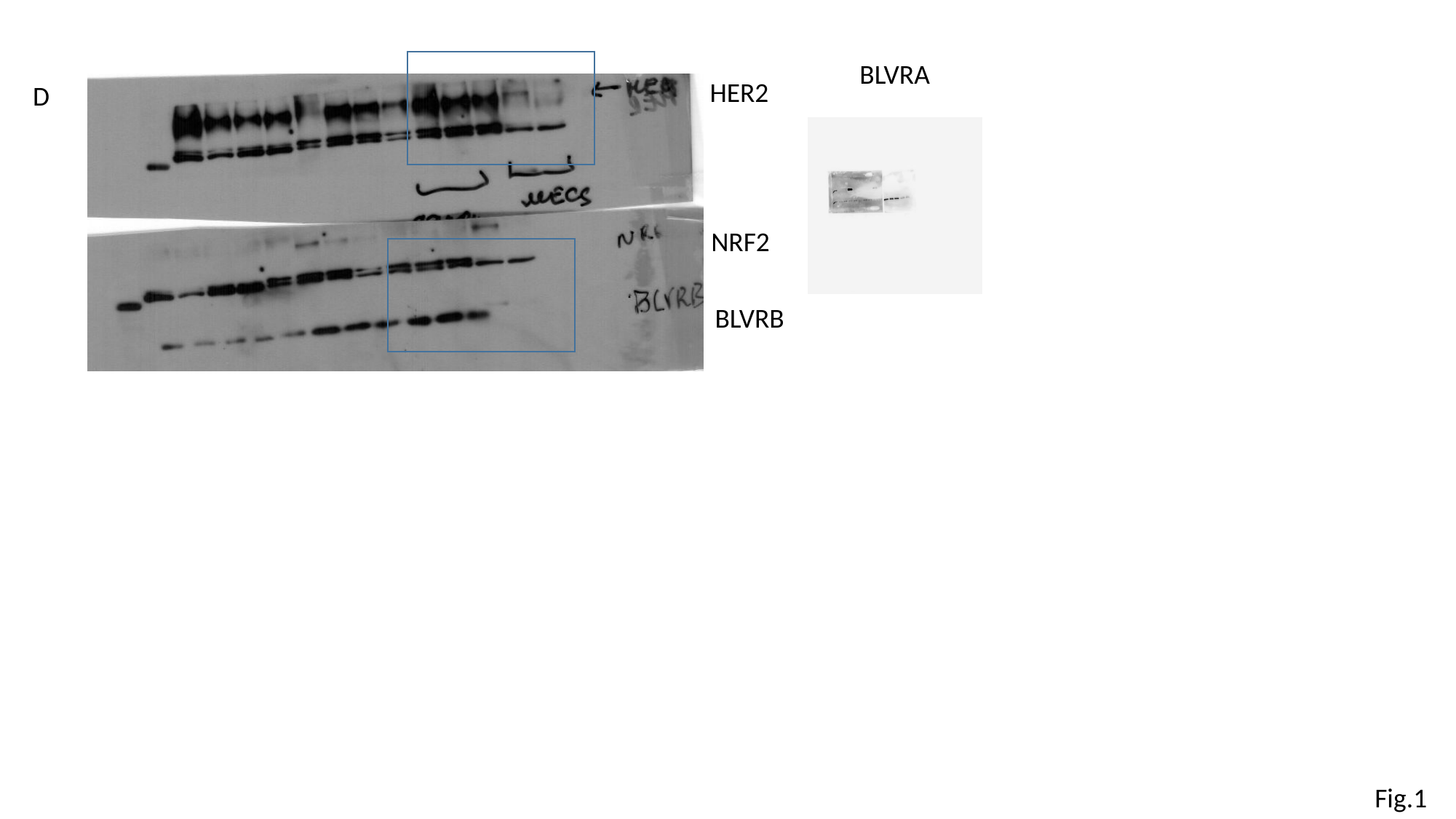

BLVRA
HER2
D
NRF2
BLVRB
Fig.1

## Slide 2
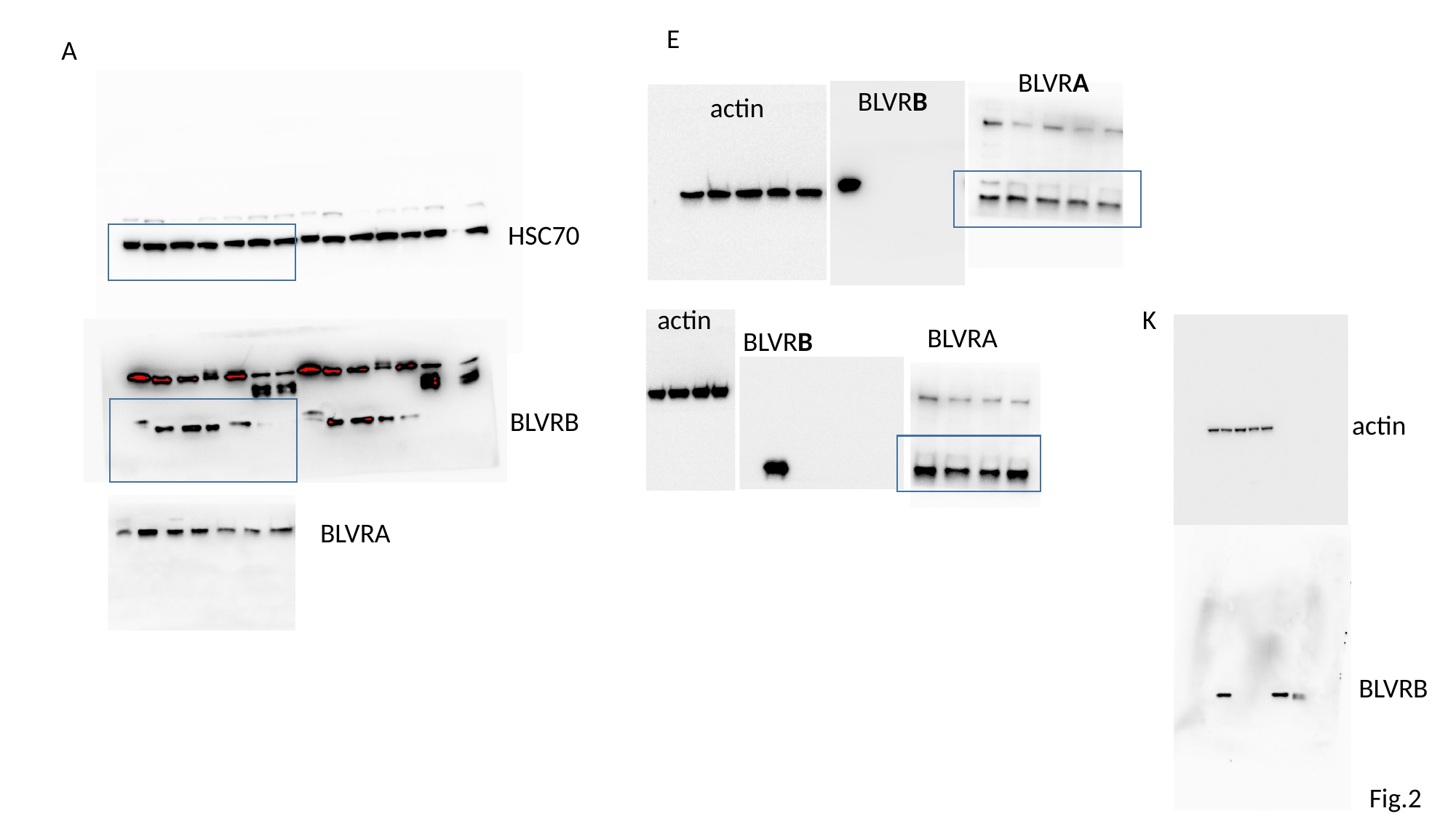

E
 A
BLVRA
BLVRB
actin
HSC70
actin
 K
BLVRA
BLVRB
BLVRB
actin
BLVRA
BLVRB
Fig.2

## Slide 3
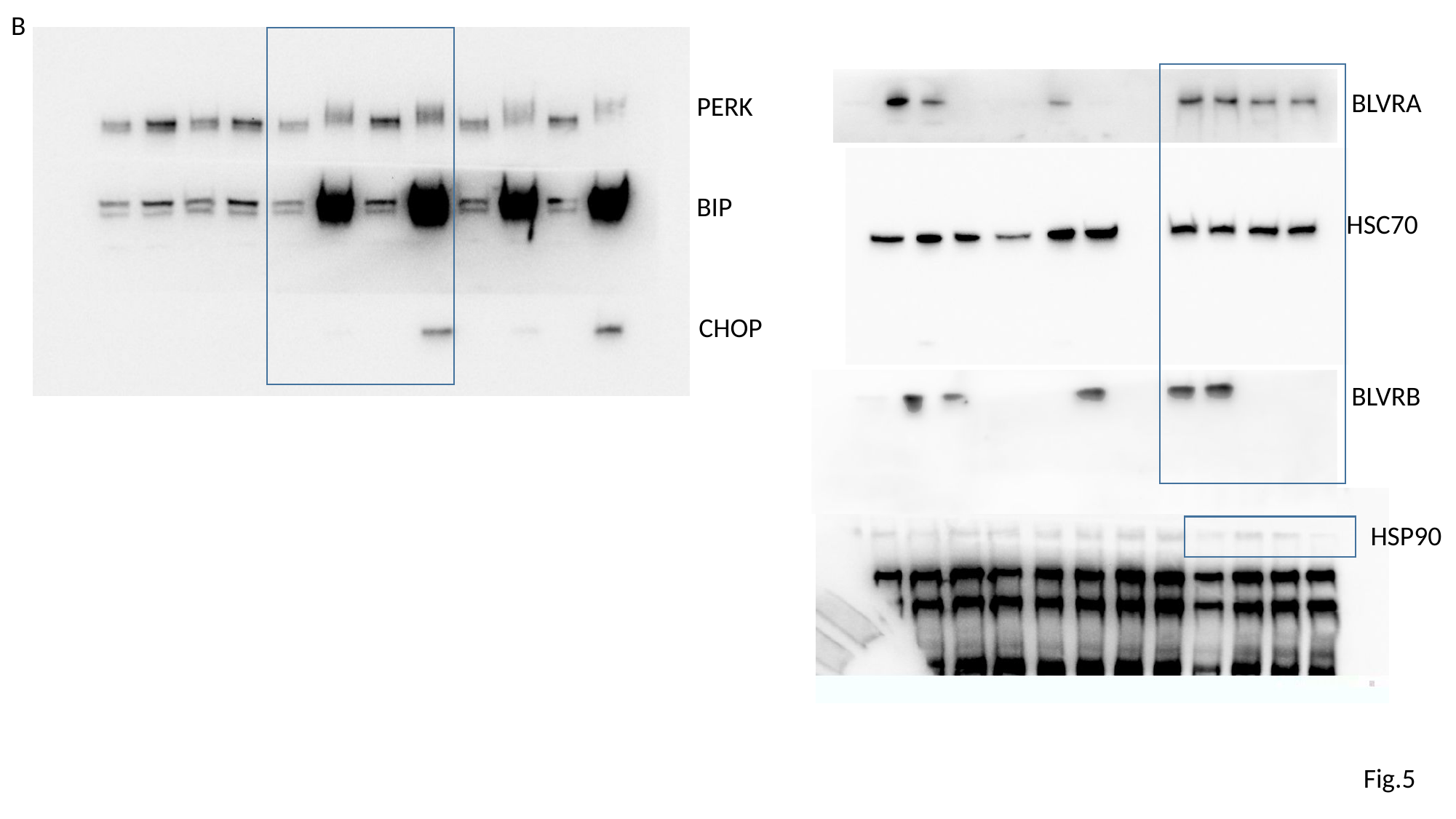

B
BLVRA
PERK
BIP
HSC70
CHOP
BLVRB
HSP90
Fig.5

## Slide 4
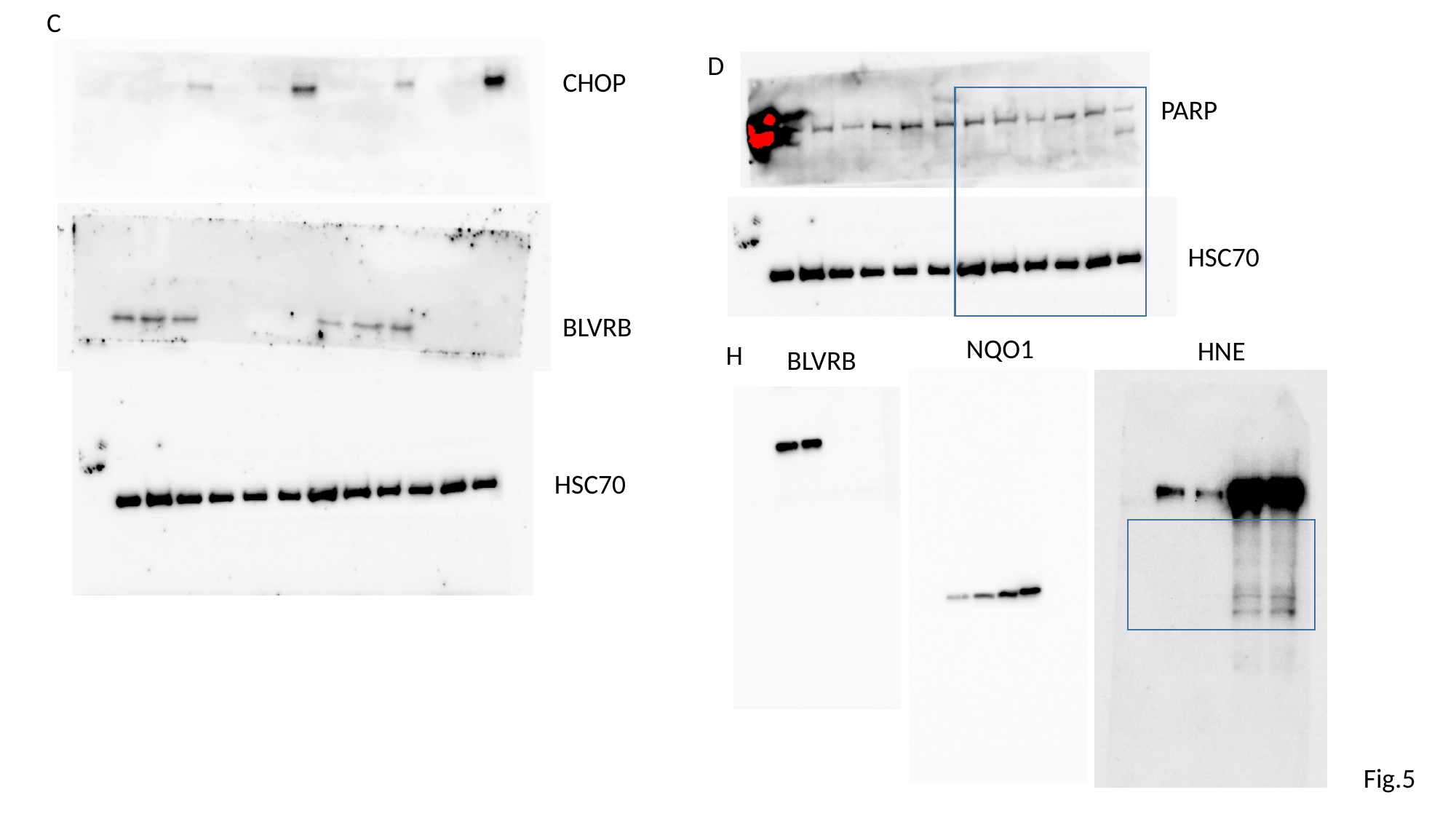

C
D
CHOP
PARP
HSC70
BLVRB
NQO1
HNE
H
BLVRB
HSC70
Fig.5

## Slide 5
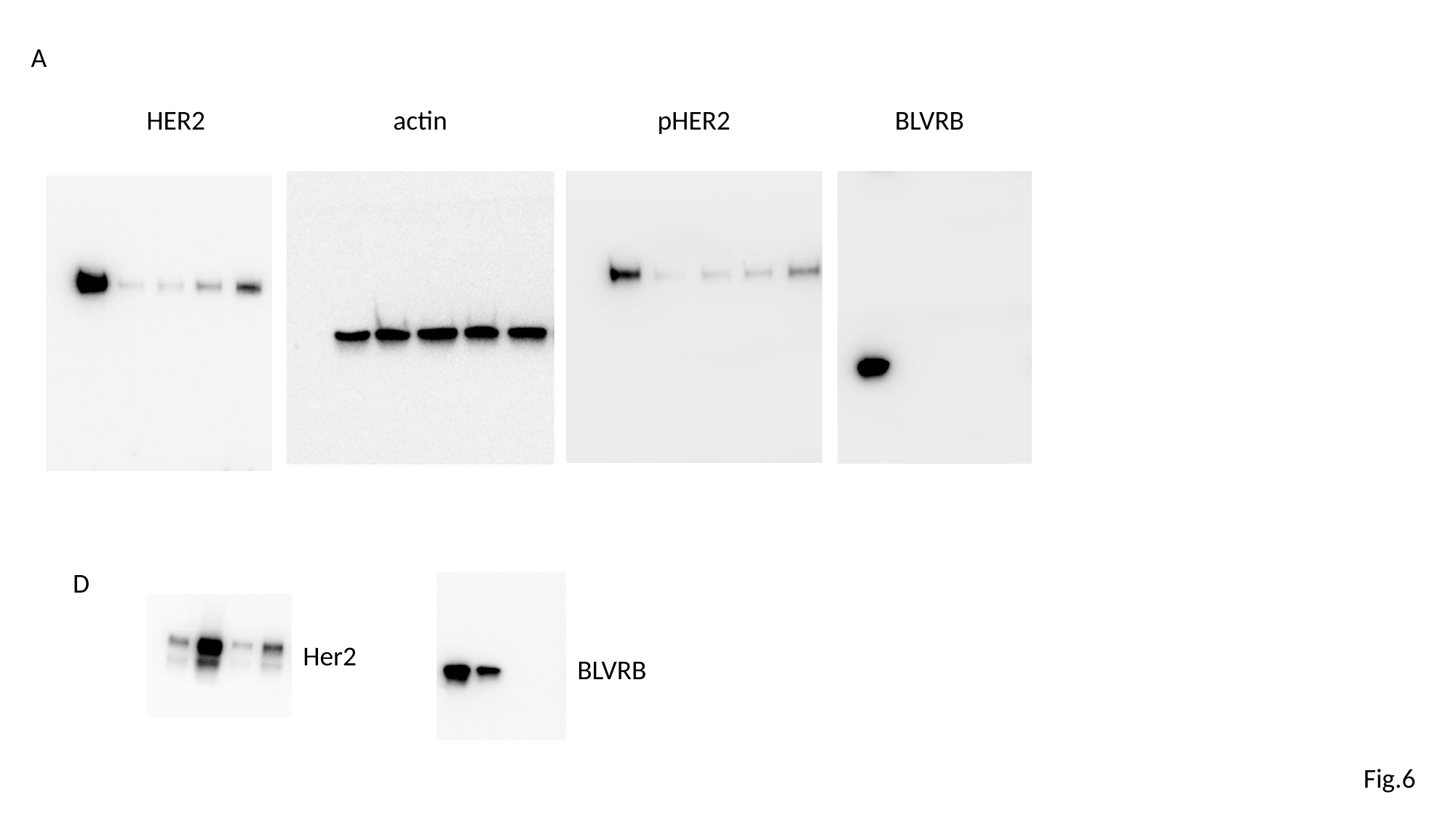

A
HER2
actin
pHER2
BLVRB
D
Her2
BLVRB
Fig.6

## Slide 6
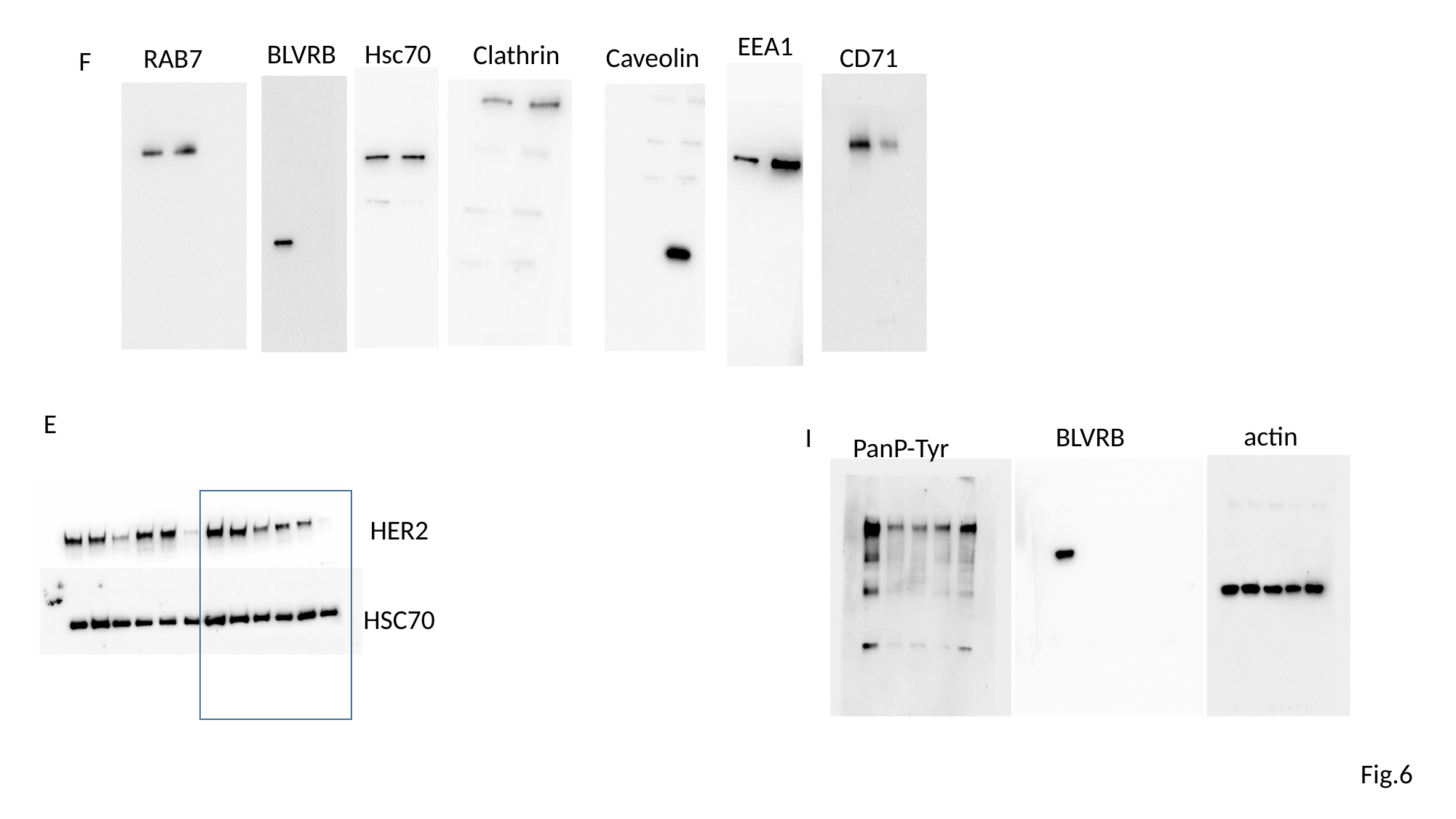

EEA1
BLVRB
Hsc70
Clathrin
Caveolin
CD71
RAB7
F
E
actin
BLVRB
I
PanP-Tyr
HER2
HSC70
Fig.6
