# Supplementary material for: Biliverdin reductase B as a new target in breast cancer
Source: Breast Cancer Res. 2025 Oct 16;27:179. doi: 10.1186/s13058-025-02147-x (PMC12532840; doi:10.1186/s13058-025-02147-x)
Supplement: Supplementary file 2 — Supplementary material 2. [file 13058_2025_2147_MOESM2_ESM.pptx]

## Slide 1
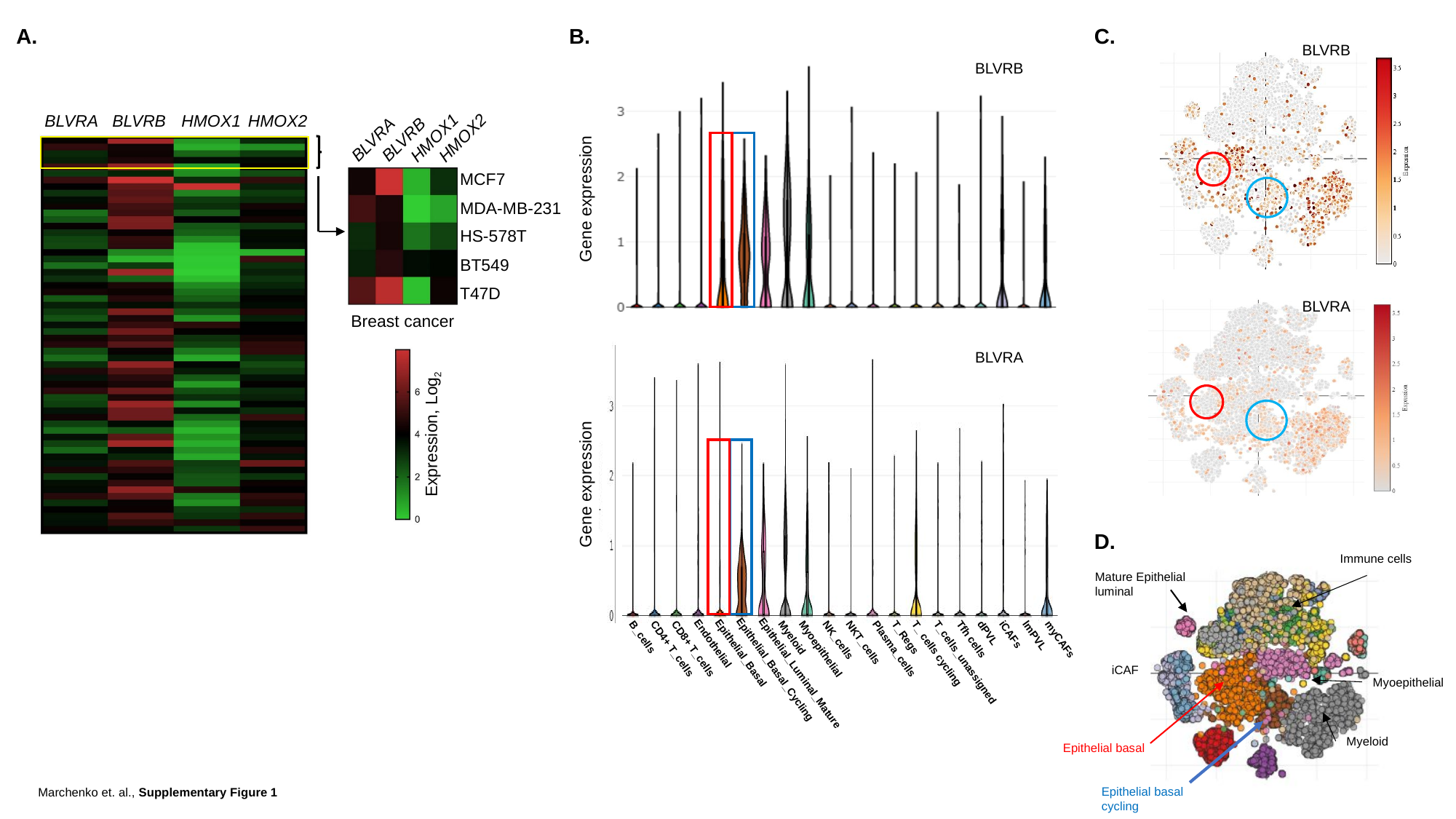

A.
B.
C.
BLVRB
BLVRB
BLVRA
BLVRB
HMOX1
HMOX2
HMOX1
HMOX2
BLVRA
BLVRB
Gene expression
MCF7
MDA-MB-231
HS-578T
BT549
T47D
BLVRA
Breast cancer
BLVRA
Expression, Log2
Gene expression
D.
Immune cells
Mature Epithelial luminal
B_cells
dPVL
iCAFs
T_Regs
Endothelial
Tfh cells
Myeloid
NK_cells
NKT_cells
lmPVL
myCAFs
Epithelial_Basal
Plasma_cells
CD4+ T_cells
CD8+ T_cells
T_ cells cycling
Myoepithelial
iCAF
Epithelial_Basal_Cycling
Epithelial_Luminal_Mature
T_cells_unassigned
Myoepithelial
Myeloid
Epithelial basal
Epithelial basal
cycling
Marchenko et. al., Supplementary Figure 1
