# Supplementary material for: Biliverdin reductase B as a new target in breast cancer
Source: Breast Cancer Res. 2025 Oct 16;27:179. doi: 10.1186/s13058-025-02147-x (PMC12532840; doi:10.1186/s13058-025-02147-x)
Supplement: Supplementary file 3 — Supplementary material 3. [file 13058_2025_2147_MOESM3_ESM.pptx]

## Slide 1
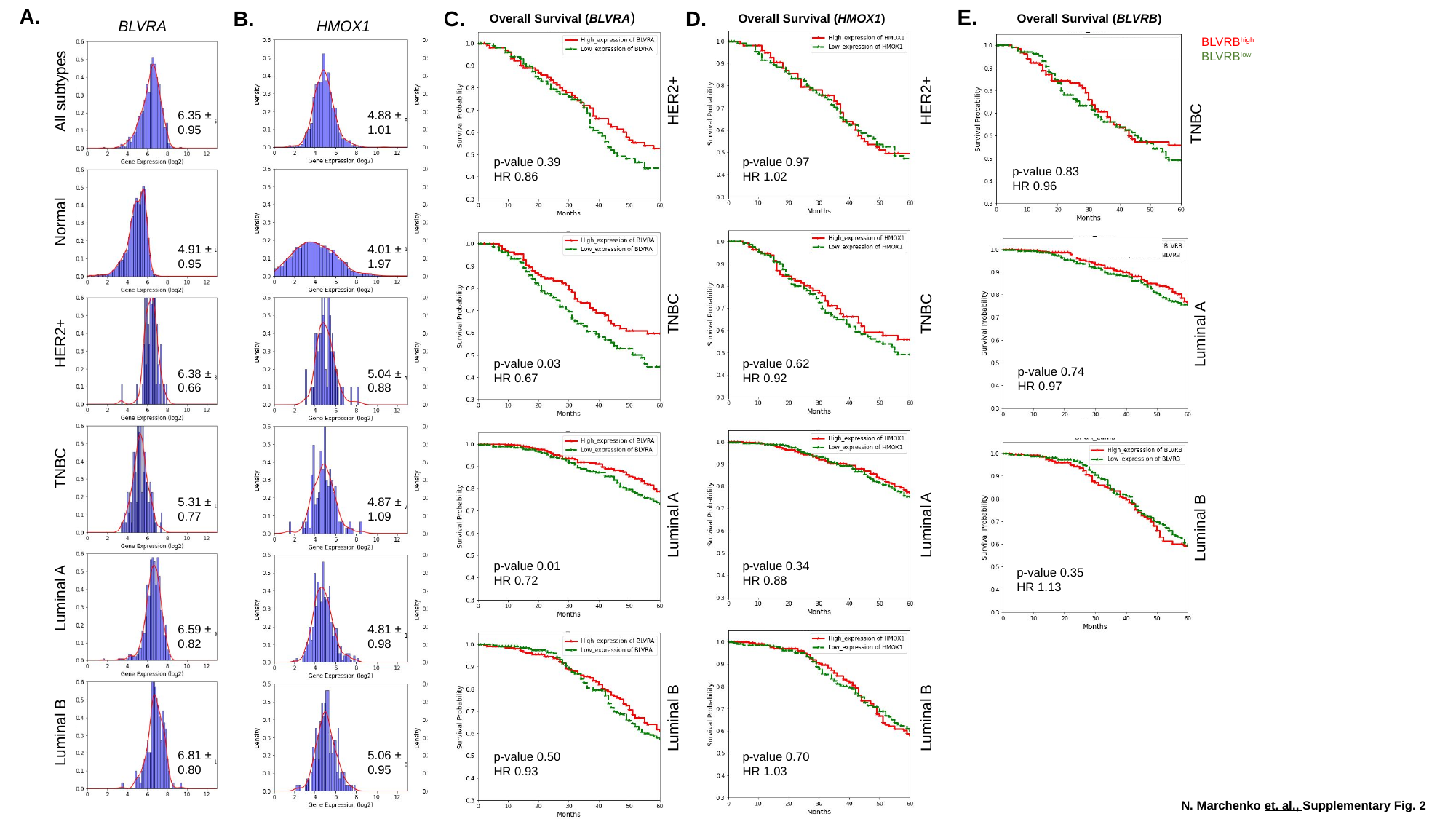

B.
D.
A.
E.
C.
Overall Survival (BLVRA)
Overall Survival (HMOX1)
Overall Survival (BLVRB)
BLVRA
HMOX1
 BLVRBhigh
 BLVRBlow
All subtypes
HER2+
HER2+
6.35 ±
0.95
4.88 ±
1.01
TNBC
p-value 0.39
HR 0.86
p-value 0.97
HR 1.02
p-value 0.83
HR 0.96
Normal
4.91 ±
0.95
4.01 ±
1.97
TNBC
TNBC
HER2+
Luminal A
p-value 0.03
HR 0.67
p-value 0.62
HR 0.92
p-value 0.74
HR 0.97
6.38 ±
0.66
5.04 ±
0.88
TNBC
5.31 ±
0.77
4.87 ±
1.09
Luminal A
Luminal A
Luminal B
p-value 0.01
HR 0.72
p-value 0.34
HR 0.88
p-value 0.35
HR 1.13
Luminal A
6.59 ±
0.82
4.81 ±
0.98
Luminal B
Luminal B
Luminal B
p-value 0.50
HR 0.93
p-value 0.70
HR 1.03
6.81 ±
0.80
5.06 ±
0.95
N. Marchenko et. al., Supplementary Fig. 2
