# Supplementary material for: Biliverdin reductase B as a new target in breast cancer
Source: Breast Cancer Res. 2025 Oct 16;27:179. doi: 10.1186/s13058-025-02147-x (PMC12532840; doi:10.1186/s13058-025-02147-x)
Supplement: Supplementary file 4 — Supplementary material 4. [file 13058_2025_2147_MOESM4_ESM.pptx]

## Slide 1
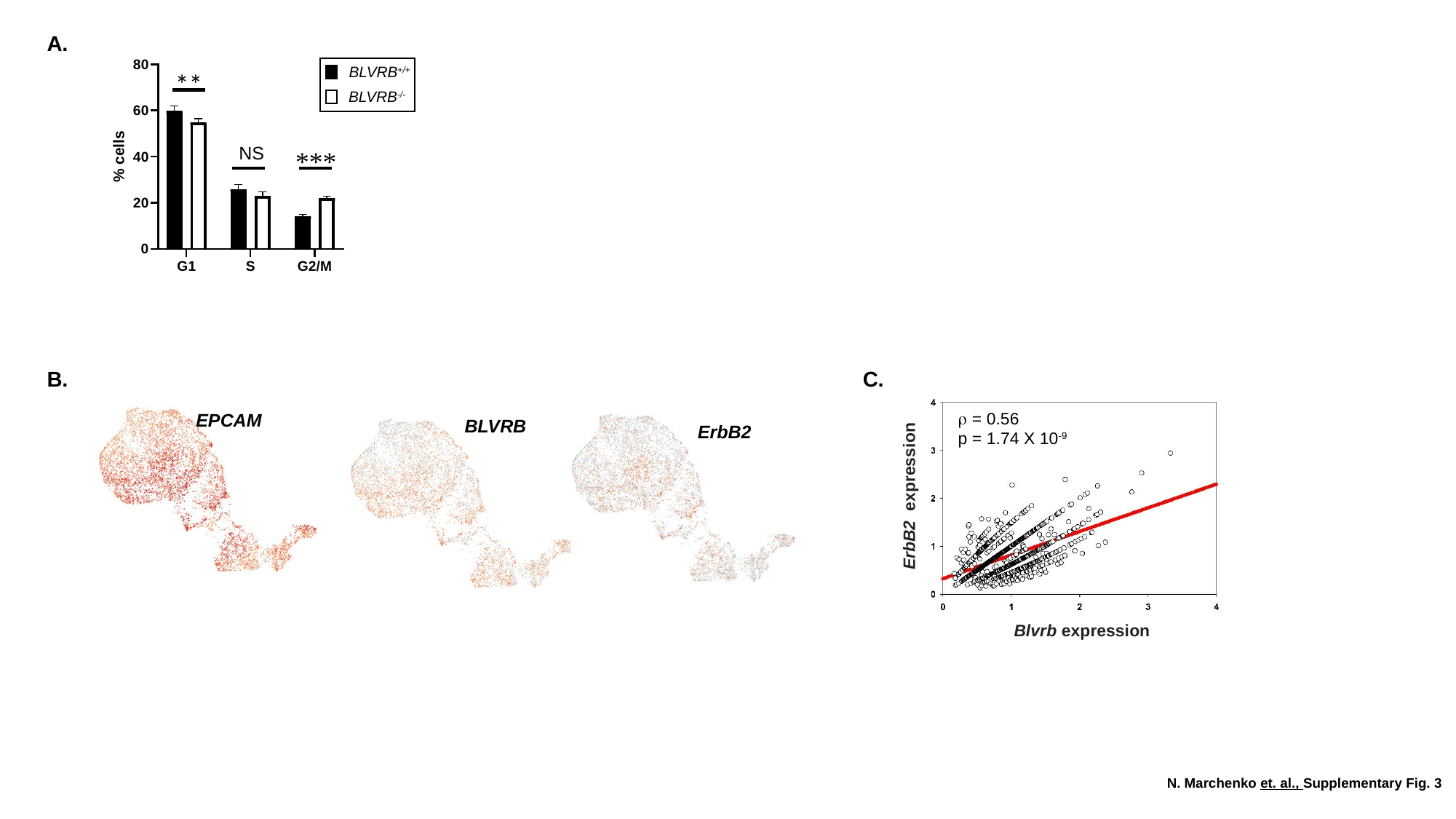

A.
BLVRB+/+
**
BLVRB-/-
NS
 ***
B.
C.
r = 0.56
p = 1.74 X 10-9
EPCAM
BLVRB
ErbB2
ErbB2 expression
Blvrb expression
N. Marchenko et. al., Supplementary Fig. 3
