# Supplementary material for: Biliverdin reductase B as a new target in breast cancer
Source: Breast Cancer Res. 2025 Oct 16;27:179. doi: 10.1186/s13058-025-02147-x (PMC12532840; doi:10.1186/s13058-025-02147-x)
Supplement: Supplementary file 5 — Supplementary material 5. [file 13058_2025_2147_MOESM5_ESM.docx]

| **Supplementary Table 1**. *Secreted metabolites (N = 138)* | | | |
| --- | --- | --- | --- |
|  | **BLVRB^-/-^/BLVRB^+/+^** | | |
| **Compound** | **Fold change (FC)** | **FC (Ratio Log_2_)** | **p-values** |
| O-dodecanoyl-carnitine (acyl-C12) | 7.65 | 2.93 | 4.04E-11 |
| L-glutamate | 6.20 | 2.63 | 1.16E-09 |
| O-tetradecanoyl-L-carnitine (acyl-C14) | 4.92 | 2.30 | 6.71E-03 |
| hexanoyl-L-carnitine (acyl-C6) | 3.50 | 1.81 | 6.59E-09 |
| Adenosine | 3.41 | 1.77 | 5.60E-04 |
| Pentanoate (valerate) | 2.82 | 1.50 | 3.80E-04 |
| Spermine | 2.67 | 1.42 | 1.43E-02 |
| butanoyl-l-carnitine (acyl-C4) | 2.64 | 1.40 | 3.97E-07 |
| acyl-C4-DC | 2.19 | 1.13 | 4.36E-07 |
| L-octanoylcarnitine (acyl-C8) | 2.00 | 1.00 | 5.78E-07 |
| Hexanoic acid (caproate) | 1.84 | 0.88 | 4.02E-04 |
| Octanoic acid (caprylate) | 1.81 | 0.86 | 3.19E-04 |
| 5-6-Dihydrothymine | 1.75 | 0.81 | 4.49E-04 |
| acetyl-carnitine (acyl-C2) | 1.67 | 0.74 | 1.01E-06 |
| 5-Hydroxyisourate | 1.62 | 0.69 | 1.05E-05 |
| (8Z-11Z-14Z)-Icosatrienoic acid | 1.55 | 0.64 | 6.73E-03 |
| 2-Hydroxyglutarate/Citramalate | 1.55 | 0.63 | 2.74E-04 |
| (S)(+)-Allantoin | 1.54 | 0.62 | 5.94E-02 |
| (5-L-Glutamyl)-L-glutamine | 1.53 | 0.62 | 6.32E-08 |
| dAMP | 1.51 | 0.59 | 3.62E-04 |
| Dodecanoic acid (lauric acid) | 1.49 | 0.57 | 5.72E-03 |
| Decanoic acid (caprate) | 1.47 | 0.56 | 1.58E-03 |
| L-glutamine | 1.46 | 0.55 | 8.10E-07 |
| Glycerol 3-phosphate | 1.45 | 0.54 | 5.04E-03 |
| 6-Hydroxykynurenic acid | 1.39 | 0.48 | 7.15E-02 |
| GDP | 1.31 | 0.39 | 2.20E-01 |
| Heptanoic acid | 1.29 | 0.37 | 8.32E-03 |
| D-Glyceraldehyde 3-phosphate/Glycerone phosphate | 1.27 | 0.34 | 3.39E-01 |
| 5-Oxoproline | 1.25 | 0.32 | 1.31E-04 |
| Itaconate | 1.25 | 0.32 | 2.22E-01 |
| GMP | 1.25 | 0.32 | 8.88E-01 |
| glycine | 1.24 | 0.31 | 8.29E-04 |
| Hypoxanthine | 1.23 | 0.30 | 7.88E-03 |
| Choline | 1.23 | 0.30 | 1.69E-02 |
| N-Acetylneuraminate | 1.22 | 0.29 | 7.50E-02 |
| 2',3'-Cyclic CMP | 1.22 | 0.28 | 8.81E-01 |
| L-serine | 1.21 | 0.27 | 1.75E-03 |
| Tetradecenoic acid (myristoleic acid) | 1.20 | 0.27 | 5.18E-01 |
| 2-Oxoglutaramate | 1.17 | 0.22 | 1.05E-01 |
| Nonanoic acid (pelargonate) | 1.14 | 0.19 | 1.37E-01 |
| L-cystine | 1.14 | 0.19 | 1.61E-02 |
| D-Glucose | 1.14 | 0.19 | 1.28E-03 |
| acyl-C20 | 1.13 | 0.18 | 8.65E-01 |
| 5-hydroxytryptophan | 1.13 | 0.18 | 5.55E-01 |
| 1-4-beta-D-Xylan | 1.11 | 0.16 | 4.64E-02 |
| L-methionine | 1.11 | 0.15 | 6.31E-04 |
| Biliverdin | 1.11 | 0.15 | 6.83E-01 |
| L-cysteine | 1.10 | 0.14 | 6.29E-02 |
| Kynurenate | 1.10 | 0.14 | 5.71E-02 |
| L-histidine | 1.10 | 0.13 | 8.39E-03 |
| D-Ribose | 1.09 | 0.12 | 2.05E-01 |
| 3-Methyleneoxindole | 1.08 | 0.12 | 2.05E-01 |
| Tryptamine | 1.08 | 0.11 | 2.01E-01 |
| Thiocysteine | 1.07 | 0.10 | 3.40E-01 |
| Nicotinamide | 1.07 | 0.09 | 7.70E-03 |
| L-tyrosine | 1.07 | 0.09 | 2.11E-02 |
| Indole | 1.06 | 0.09 | 9.95E-01 |
| Hexadecenoic acid (Palmitoleic acid) | 1.06 | 0.09 | 6.57E-01 |
| L-threonine | 1.06 | 0.08 | 1.10E-02 |
| 2-Oxoadipate | 1.06 | 0.08 | 6.43E-01 |
| Pantothenate | 1.05 | 0.07 | 5.12E-01 |
| L-lysine | 1.04 | 0.06 | 1.16E-01 |
| Pyridoxal | 1.04 | 0.06 | 4.49E-01 |
| L-asparagine | 1.04 | 0.06 | 7.94E-01 |
| L-phenylalanine | 1.04 | 0.05 | 1.58E-02 |
| Diphosphate | 1.04 | 0.05 | 7.35E-01 |
| Guanine | 1.03 | 0.04 | 1.85E-01 |
| Docosahexaenoic acid | 1.02 | 0.03 | 7.84E-01 |
| N-Succinyl-L-glutamate 5-semialdehyde | 1.02 | 0.03 | 2.50E-01 |
| Lactate | 1.02 | 0.03 | 6.92E-01 |
| Pyridoxamine 5'-phosphate | 1.01 | 0.02 | 4.93E-01 |
| L-tryptophan | 1.01 | 0.02 | 4.51E-01 |
| Nicotinate ribonucleotide | 1.01 | 0.01 | 9.50E-01 |
| Bis-gamma-glutamylcystine | 1.01 | 0.01 | 9.13E-01 |
| Folate | 1.01 | 0.01 | 8.06E-01 |
| L-valine | 1.00 | 0.01 | 3.33E-01 |
| Ornithine | 1.00 | 0.00 | 7.32E-01 |
| Sphingosine | 0.99 | -0.01 | 2.88E-01 |
| Tetradecanoic acid (myristic acid) | 0.99 | -0.02 | 5.84E-01 |
| Hexadecanoic acid (palmitic acid) | 0.99 | -0.02 | 8.42E-01 |
| L-arginine | 0.99 | -0.02 | 9.66E-01 |
| IDP | 0.98 | -0.03 | 8.64E-01 |
| O-dodecenoyl-carnitine (acyl-C12:1) | 0.98 | -0.03 | 3.34E-01 |
| UMP | 0.98 | -0.04 | 5.09E-01 |
| Indole-3-acetate | 0.97 | -0.04 | 1.91E-01 |
| UDP | 0.97 | -0.04 | 6.10E-01 |
| ADP-D-ribose | 0.97 | -0.05 | 7.52E-01 |
| O-Decenoyl-L-carnitine (acyl-C10:1) | 0.96 | -0.05 | 3.61E-01 |
| L-leucine/isoleucine | 0.95 | -0.07 | 5.28E-01 |
| Phosphocreatine | 0.95 | -0.08 | 6.54E-01 |
| Mannitol | 0.94 | -0.09 | 1.84E-01 |
| 2-Aminomuconate | 0.94 | -0.09 | 5.86E-01 |
| 5'-Phosphoribosyl-N-formylglycinamide | 0.93 | -0.10 | 1.06E-01 |
| L-Methionine S-oxide | 0.93 | -0.11 | 1.66E-01 |
| Acetylcholine | 0.93 | -0.11 | 1.51E-01 |
| Hydroxyindole-acetylglycine | 0.92 | -0.12 | 3.05E-01 |
| Indolepyruvate | 0.92 | -0.13 | 3.41E-01 |
| Octadecenoic acid (Oleic acid) | 0.90 | -0.15 | 2.96E-01 |
| Dodecanedioic acid | 0.89 | -0.18 | 2.31E-01 |
| gamma-Glutamyl-gamma-aminobutyrate | 0.89 | -0.18 | 7.74E-02 |
| 2-Oxoglutarate | 0.88 | -0.18 | 2.94E-02 |
| g-Oxalo-crotonate | 0.87 | -0.21 | 3.68E-01 |
| 5-Phosphoribosylamine | 0.81 | -0.31 | 6.25E-01 |
| Creatine | 0.81 | -0.31 | 9.61E-03 |
| Indoxyl | 0.80 | -0.33 | 8.11E-02 |
| N-Methylethanolamine phosphate | 0.79 | -0.34 | 5.68E-02 |
| Indole-3-acetaldehyde | 0.78 | -0.36 | 4.44E-04 |
| L-Carnitine | 0.77 | -0.39 | 2.26E-02 |
| Linoleic acid ((9Z,12Z)-Octadecadienoic acid) | 0.75 | -0.41 | 2.74E-01 |
| O-octadecenoyl-L-carnitine (acyl-C18:1) | 0.75 | -0.42 | 6.41E-01 |
| L-alanine | 0.74 | -0.44 | 2.74E-03 |
| Creatinine | 0.74 | -0.44 | 1.17E-02 |
| Maltotriose | 0.73 | -0.46 | 3.81E-02 |
| Xanthine | 0.71 | -0.49 | 2.06E-02 |
| Anthranilate | 0.71 | -0.50 | 9.27E-03 |
| Dehydroascorbate | 0.70 | -0.51 | 1.99E-02 |
| gamma-L-Glutamyl-D-alanine | 0.70 | -0.51 | 5.83E-02 |
| IMP | 0.67 | -0.57 | 6.26E-01 |
| Bilirubin | 0.66 | -0.61 | 1.15E-01 |
| L-aspartate | 0.64 | -0.64 | 2.12E-01 |
| Arachidonic acid (Eicosatetraenoic acid) | 0.64 | -0.64 | 1.71E-03 |
| L-proline | 0.62 | -0.69 | 1.03E-05 |
| L-Palmitoylcarnitine (acyl-C16) | 0.59 | -0.75 | 2.56E-01 |
| Phosphate | 0.57 | -0.82 | 5.25E-03 |
| (R)-S-Lactoylglutathione | 0.46 | -1.13 | 7.76E-02 |
| Octadecanoyl-L-carnitine (acyl-C18) | 0.46 | -1.14 | 2.45E-01 |
| Succinate | 0.49 | -1.03 | 7.71E-06 |
| Citrate | 0.45 | -1.15 | 8.31E-05 |
| Inosine | 0.41 | -1.28 | 5.72E-07 |
| Pyruvate | 0.36 | -1.49 | 2.78E-09 |
| Malate | 0.33 | -1.61 | 2.58E-07 |
| Fumarate | 0.33 | -1.61 | 4.24E-08 |
| Ascorbate | 0.30 | -1.74 | 7.71E-07 |
| propionyl-carnitine (acyl-C3) | 0.18 | -2.47 | 3.66E-05 |
| D-Glucose 6-phosphate | 0.12 | -3.05 | 2.10E-06 |
| Uracil | 0.10 | -3.38 | 1.19E-05 |
| D-Fructose 1-6-bisphosphate | 0.09 | -3.55 | 4.41E-07 |
| Allantoate | 0.03 | -4.88 | 5.72E-09 |

†Upregulated (BLVRB^-/-^/BLVRB^+/+^) metabolites with Log2 FC ≥ 1, p-value ≤ 0.01 highlighted green (**N = 10**)

Downregulated (BLVRB^-/-^/BLVRB^+/+^) metabolites with Log2 FC ≤ 1, p-value ≤ 0.01 highlighted blue (**N = 12**)
