# Supplementary material for: Biliverdin reductase B as a new target in breast cancer
Source: Breast Cancer Res. 2025 Oct 16;27:179. doi: 10.1186/s13058-025-02147-x (PMC12532840; doi:10.1186/s13058-025-02147-x)
Supplement: Supplementary file 6 — Supplementary material 6. [file 13058_2025_2147_MOESM6_ESM.docx]

| **Supplementary Table 2.** *Oligonucleotide primers* | | | |
| --- | --- | --- | --- |
| **Gene description** | **Gene symbol** | **Forward primer (5’ to 3’)** | **Reverse primer (5’ to 3’)** |
|  |  |  |  |
| Actin, beta | *ACTB* | TACCACAGGCATTGTGATGG | TTTGATGTCACGCACGATTT |
| ErbB2 Receptor Tyrosine Kinase 2 | *ERBB2* | GGAAGTACACGATGCGGAGACT | ACCTTCCTCAGCTCCGTCTCTT |
